# Metabolic profiling of sourdough fermented wheat and rye bread

**DOI:** 10.1038/s41598-018-24149-w

**Published:** 2018-04-09

**Authors:** Ville M. Koistinen, Outi Mattila, Kati Katina, Kaisa Poutanen, Anna-Marja Aura, Kati Hanhineva

**Affiliations:** 1 University of Eastern Finland, Institute of Public Health and Clinical Nutrition, P.O. Box 1627, FI-70211 Kuopio, Finland; 2 VTT Technical Research Centre of Finland, P.O. Box 1000, Tietotie 2, Espoo, FI-02044 VTT Finland; 3 University of Helsinki, Department of Food and Environmental Sciences, P.O. Box 66, FI-00014 Helsinki, Finland

## Abstract

Sourdough fermentation by lactic acid bacteria is commonly used in bread baking, affecting several attributes of the final product. We analyzed whole-grain wheat and rye breads and doughs prepared with baker’s yeast or a sourdough starter including *Candida milleri*, *Lactobacillus brevis* and *Lactobacillus plantarum* using non-targeted metabolic profiling utilizing LC–QTOF–MS. The aim was to determine the fermentation-induced changes in metabolites potentially contributing to the health-promoting properties of whole-grain wheat and rye. Overall, we identified 118 compounds with significantly increased levels in sourdough, including branched-chain amino acids (BCAAs) and their metabolites, small peptides with high proportion of BCAAs, microbial metabolites of phenolic acids and several other potentially bioactive compounds. We also identified 69 compounds with significantly decreased levels, including phenolic acid precursors, nucleosides, and nucleobases. Intensive sourdough fermentation had a higher impact on the metabolite profile of whole-grain rye compared to milder whole-grain wheat sourdough fermentation. We hypothesize that the increased amount of BCAAs and potentially bioactive small peptides may contribute to the insulin response of rye bread, and in more general, the overall protective effect against T2DM and CVD.

## Introduction

Increasing evidence is supporting the protective effect of whole-grain cereal consumption against several noncommunicable diseases, such as type 2 diabetes mellitus, cardiovascular disease and colorectal cancer, as well as overall mortality^1–3^. This has been attributed to cereal dietary fiber and the array of phytochemicals within the fiber matrix^4,5^, both of which interact with gastrointestinal microbiota and undergo transformations, possibly mediating physiological changes^6^. However, the metabolic pathways leading to these effects are still mostly unknown. Among the phytochemical classes, phenolic acids and alkylresorcinols are abundant in the bran section of whole grains^7,8^. These compounds have shown antioxidative, antimicrobial and anticancer effects *in vitro*.

Bread is one of the most important staple foods consumed worldwide and thus serves as a major source of whole-grain cereals^9^. The intake of bread baked from whole-grain rye (*Secale cereale* L.) has been shown to cause a lowered postprandial insulin response, known as the rye factor^10,11^, via unknown mechanisms. Common wheat (*Triticum aestivum* L.) is one of the primary sources of dietary fiber in the United States and several other industrialized countries, whereas rye provides an important dietary fiber source in parts of Northern and Eastern Europe. In the baking of nearly all rye breads and several artisanal wheat breads, such as the San Francisco bread, sourdough fermentation by lactic acid bacteria (LAB) and yeasts is used to improve the texture, sensory properties and shelf life of the bread product^12^. Whereas the emergence of industrial baking caused yeast fermentation to become the dominant practice in bread baking, sourdough has gained interest during the last years, not only from the technical and gastronomical perspective but also because of the increased nutritional value and potential health benefits offered by the ancient biotechnological process^13^. Sourdough fermentation is known to cause transformations of lipids and macromolecules^14^ and several phytochemicals, such as phenolic acids, folates and sugar-conjugated bioactive compounds^15^. However, the overall metabolism of small molecules during sourdough fermentation has not been studied with a non-targeted method or any other comprehensive chemical analysis.

The aim of this study was to elucidate the changes in the metabolite profile caused by sourdough fermentation as compared to yeast fermentation and to indirectly compare the differences between sourdough-fermented wholegrain wheat and rye breads. Emphasis was given to bioactive compounds, which potentially contribute to the health benefits of whole-grain products. Similar starters, containing *Candida milleri*, *Lactobacillus brevis* and *Lactobacillus plantarum*, were selected for both types of doughs to represent a typical microbial distribution in sourdough. The fermentation conditions for wheat and rye were chosen to represent a typical sourdough process for each cereal. Liquid chromatography–mass spectrometry (LC–MS) -based non-targeted metabolomics was used to detect and identify the discriminatory compounds for each comparison. The proportion of identified compounds was maximized by combining the use of a standard library, database searches, *in silico* generated mass spectra, and MS/MS fragment motifs associated with certain molecular moieties.

## Results

### Effect of sourdough on the metabolic profiles

**Figure 1:**
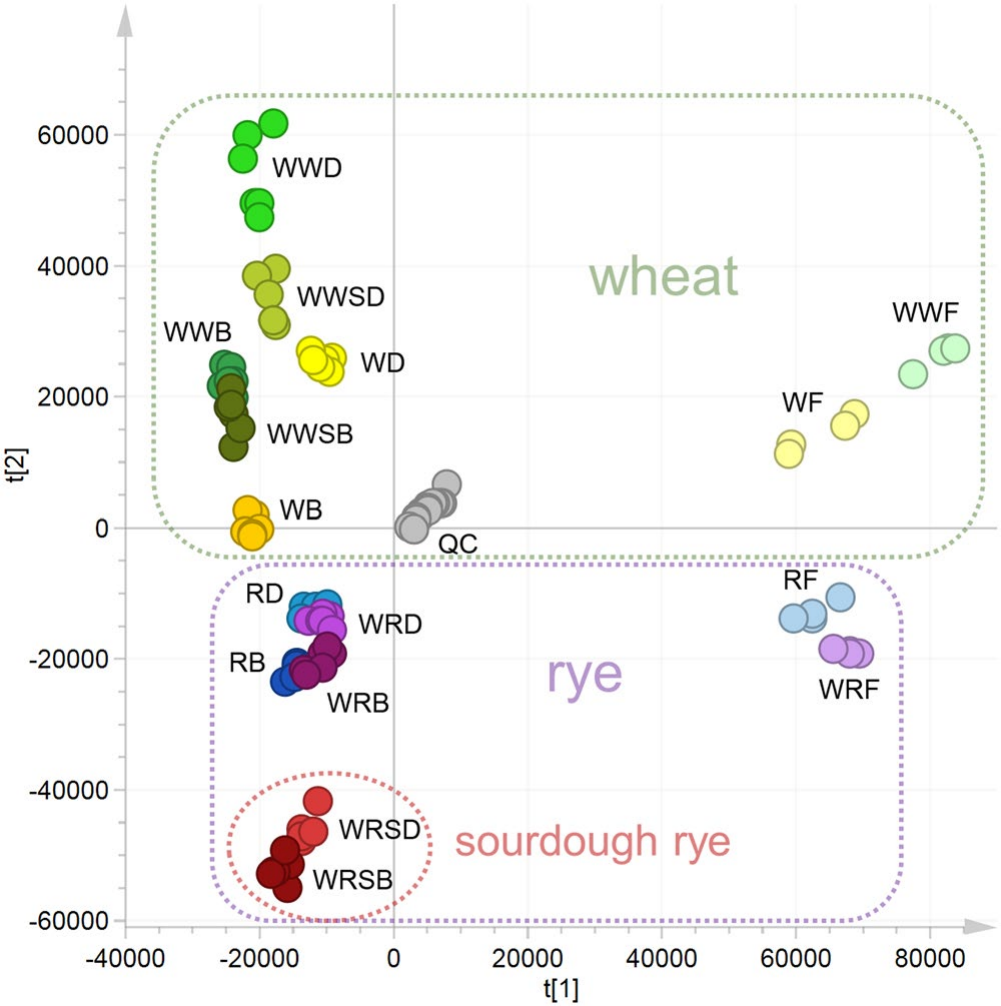
Principal component analysis (PCA) of the bread samples. This figure contains the first two principal components and their scores t1 and t2, which explain 21% and 16% of the variation within the data, respectively. WF = white wheat flour, WWF = whole-grain wheat flour, RF = refined rye flour, WRF = whole-grain rye flour, WD = white wheat dough, WB = white wheat bread, WWD = whole-grain wheat dough, WWB = whole-grain wheat bread, WWSD = whole-grain wheat sourdough, WWSB = whole-grain wheat sourdough bread, RD = refined rye dough, RB = refined rye bread, WRD = whole-grain rye dough, WRB = whole-grain rye bread, WRSD = whole-grain rye sourdough, WRSB = whole-grain rye sourdough bread, QC = quality control from pooled samples.

Principal component analysis (PCA) of the raw data, including the two first components and all the detected molecular features, showed a clear separation of the metabolic profiles of the flours from the rest of the sample groups (doughs and breads) along the first component of the PCA (Fig. 1). It also revealed a pronounced separation of the sourdough fermented whole-grain rye bread (WWSB) and dough (WWSD) from the other sample groups along the second component. In contrast, yeast fermented whole-grain rye bread and dough showed no major difference to similarly prepared bread and dough produced from sifted rye flour (with bran section removed). Sourdough fermented whole-grain wheat bread and dough, however, did not show as large changes in their metabolite profiles as rye when compared with their yeast fermented counterparts. In wheat, the difference between the overall metabolite profiles depended mostly on the whole-grain content of the samples. The changes in the profiles occur mostly during the baking of the dough, as indicated by the proximity of the dough and bread samples of similar bread types in the PCA.

### Metabolites with increased levels

**Figure 2:**
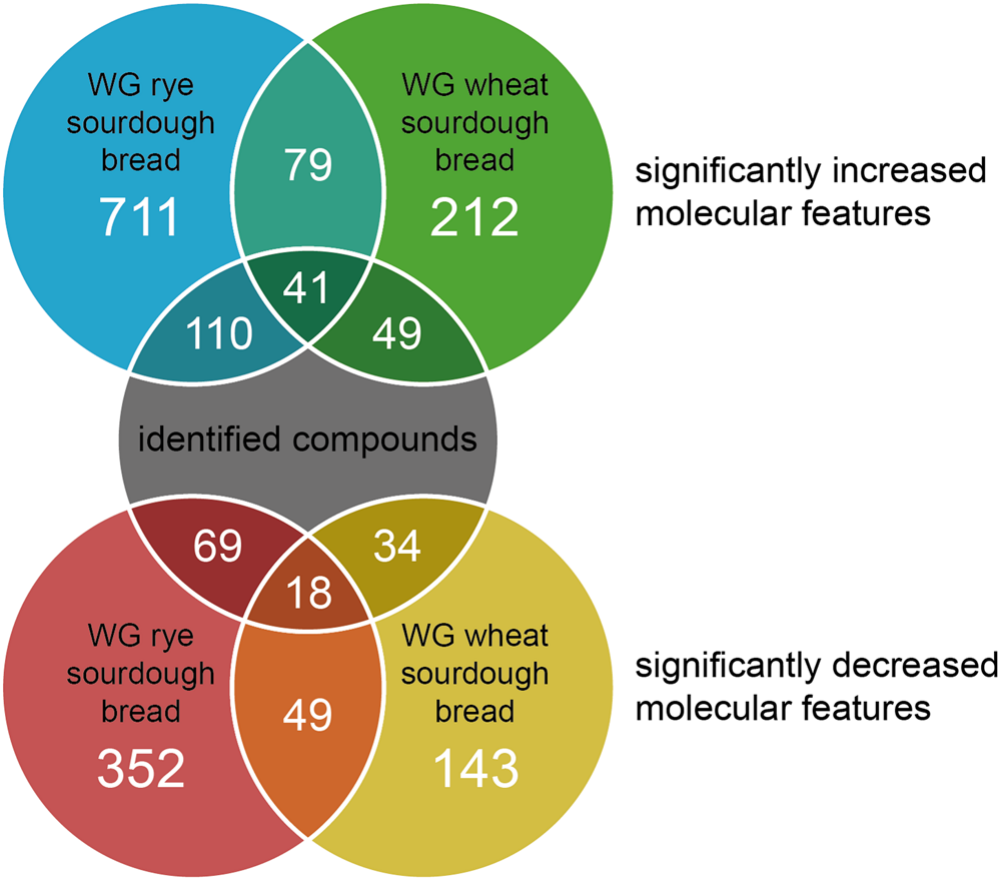
The number of molecular features and identified compounds with significantly increased or decreased levels in the whole-grain sourdough rye and wheat breads compared to their yeast-fermented counterparts.

**Figure 3:**
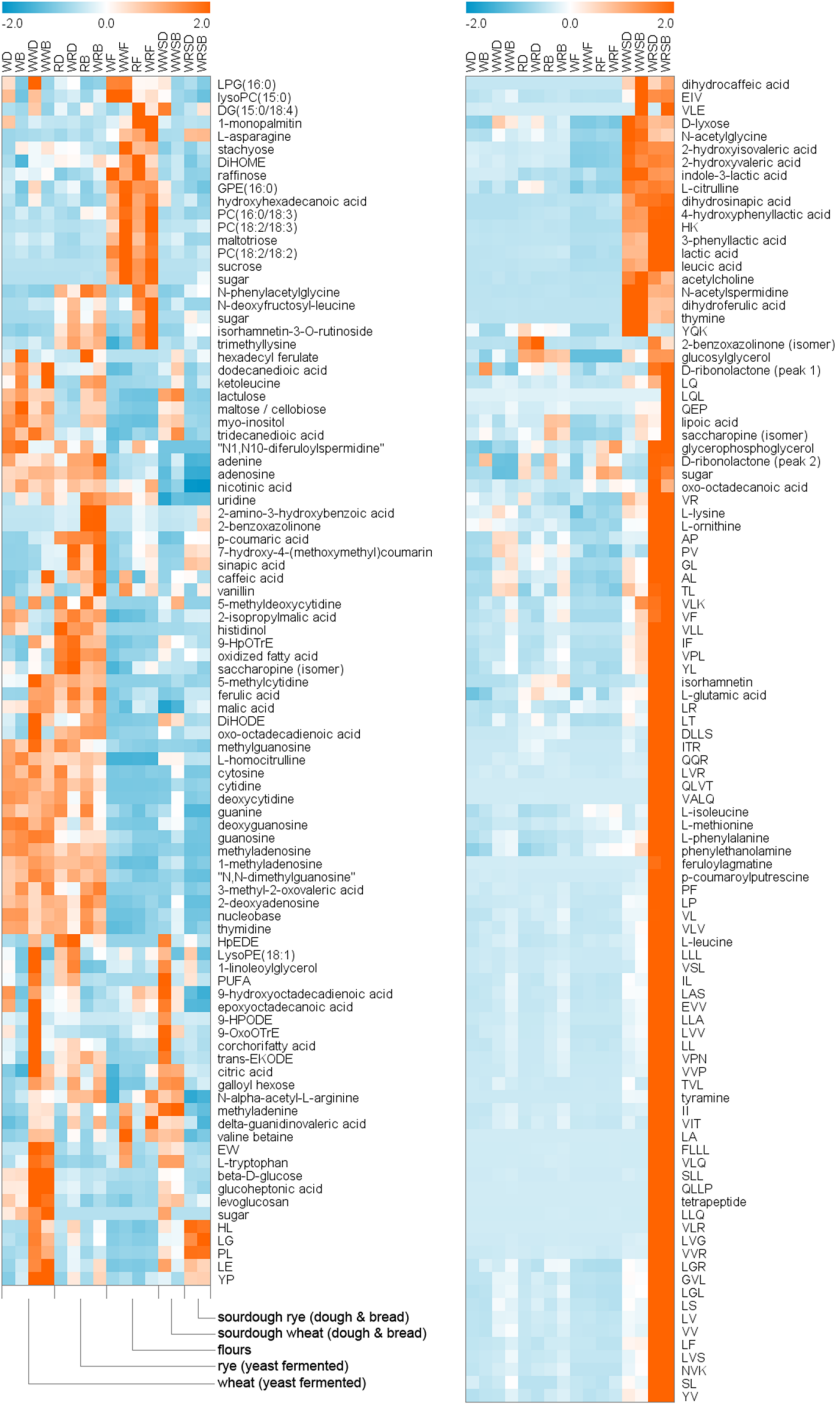
Heat map of the identified compounds with significant level increase (orange) or decrease (blue) (*p* < 0.01, FC ≥ 2) in sourdough wheat and/or rye bread. Data is included from all the studied samples.

When comparing data from sourdough and yeast fermented whole-grain rye breads, 711 molecular features fulfilled the criteria of significant increase in sourdough rye bread (*p* < 0.01, fold change (FC) ≥ 2). Correspondingly, 212 features fulfilled the same criteria in the sourdough fermented wheat bread. Out of these, 79 features had significantly increased levels in both cereal type sourdough breads (Fig. 2). After the identification process and removal of redundant ions from the data from different ionization modes and columns, 118 distinct compounds were identified with a standard or putatively identified among the significant compounds, out of which 110 were significant in the rye bread comparison (sourdough vs. yeast fermented), 49 in the wheat bread comparison, and 41 in both comparisons. Table S1 shows the identified compounds along with their identification and statistical data. The identified compounds and their relative levels across all studied samples were visualized in a heat map (Fig. 3).

**Figure 4:**
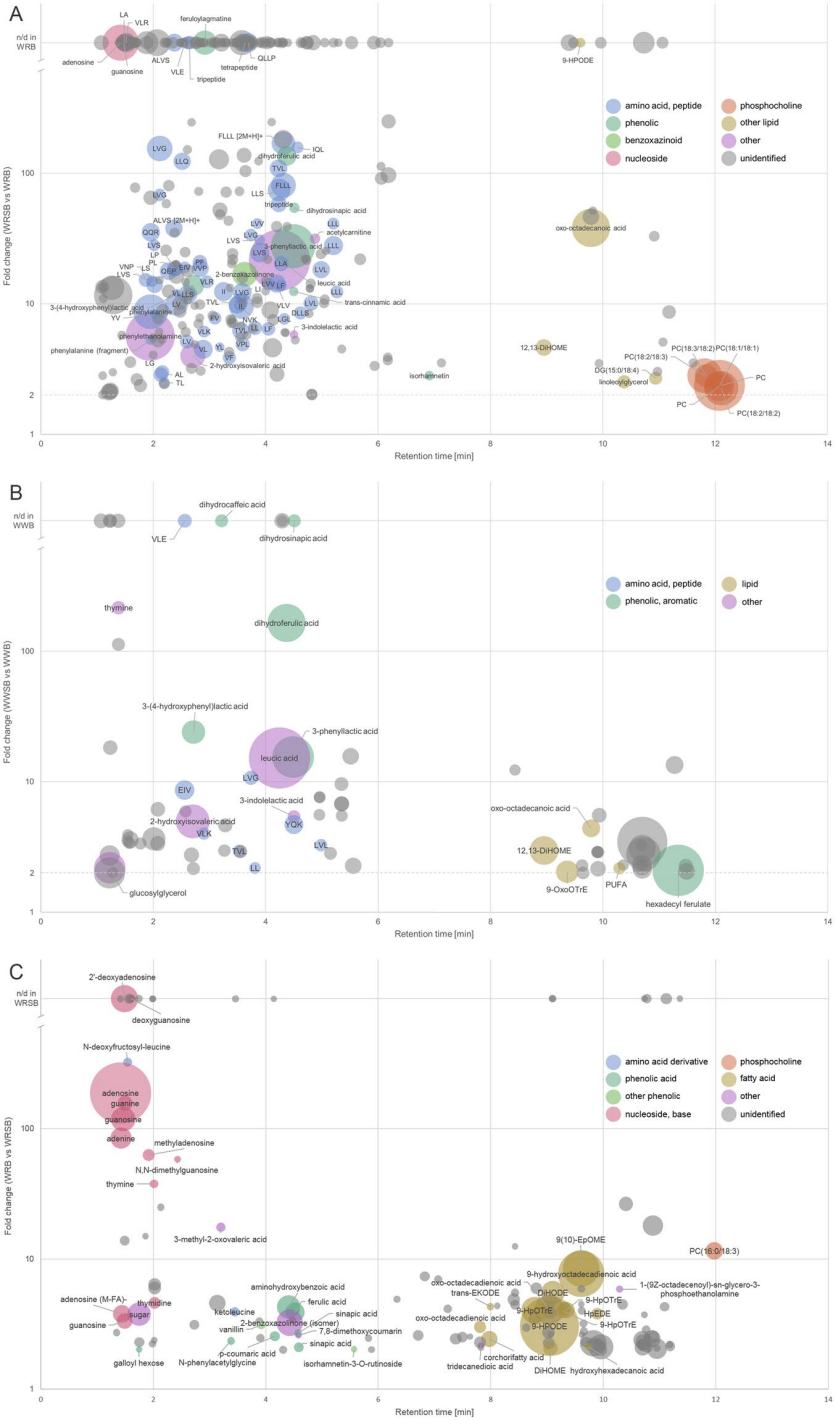
ubble plots of the statistically significant (*p* < 0.01) compounds with increased levels (FC ≥ 2) in sourdough whole-grain rye breads compared with corresponding yeast fermented whole-grain breads. The area of each sphere represents the average signal intensity of the molecular ion in the sourdough fermented bread and the color represents the compound group. Unidentified compounds are grey in color. The y-axis (fold change) has been transformed into base-10 logarithmic scale, which has been truncated to show the compounds with an infinite fold change (compounds not detected in yeast fermented bread). (**A**) Increased compounds in sourdough whole-grain rye bread detected with the reversed-phase (RP) column in the positive and negative mode. (**B**) Increased compounds in sourdough whole-grain wheat bread detected with the RP column in the positive and negative mode. (**C**) Compounds with significantly decreased levels in sourdough whole-grain rye bread detected with the RP column in the positive and negative mode.

Overall, the identified differential compounds of sourdough fermentation fell into several categories of potentially bioactive chemicals. In sourdough rye, the most notable group of identified compounds was amino acids and their derivatives, including metabolites of amino acids and di-, tri- and tetrapeptides (Table S1). The amino acids included eight proteinogenic amino acids (asparagine, glutamic acid, isoleucine, leucine, lysine, phenylalanine, methionine, and tryptophan) and four other amino acids (citrulline, homocitrulline, ornithine, and saccharopine). Notably, two branched-chain amino acids (BCAA), leucine and isoleucine, as well as leucic acid (2-hydroxyisocaproic acid), a metabolite of leucine, were among the differential compounds. The increase in the levels of leucine and isoleucine was 17-fold and 10-fold, respectively. The increased metabolites of amino acids also included 2-hydroxyisovaleric acid (from BCAAs), tryptophan metabolites indole-3-lactic acid and 3-phenyllactic acid, and tyrosine metabolites 4-hydroxyphenyllactic acid and tyramine. Out of the 70 small peptides putatively identified, 64 fulfilled the significance criteria in rye and 62 contained one or several BCAA residues. As indicated by the heat map (Fig. 3), most of the small peptides and several amino acids and their metabolites were highly specific to whole-grain rye sourdough and the corresponding bread. Three microbial metabolites of phenolic acids (dihydroferulic acid, dihydrocaffeic acid and dihydrosinapic acid), phenolic acid derivatives (feruloylagmatine and *p*-coumaroylputrescine), six sugars or sugar derivatives, two phosphatidylcholines [PC(18:2/18:2) and PC(18:2/18:3)], and two fatty acids were among the increased compounds. Other identified potentially bioactive compounds with significantly increased levels were 2-benzoxazolinone (benzoxazinoid), 2-hydroxyvaleric acid, isorhamnetin (flavonoid), *N*-acetylspermidine, and phenylethanolamine. The identified and unidentified compounds were plotted based on their retention time, fold change and average signal intensity to highlight the most important differential compounds found in the analysis (Fig. 4).

In wheat sourdough, two amino acids (citrulline and saccharopine), 29 small peptides and six amino acid metabolites (the same as in rye sourdough) had significantly increased levels (*p* <  < 0.01, FC ≥ 2) (Fig. 4B). In contrast to rye, none of the proteinogenic amino acids were among the differential compounds. One small peptide, YQK, was significantly increased only in wheat sourdough. Phenolic acids and their microbial metabolites behaved similarly in wheat sourdough compared to rye (Fig. 3). No phosphatidylcholines were significantly changed in wheat.

### Decreased and unchanged metabolites

We also investigated the compounds that had significantly decreased levels in the sourdough bread samples. In the rye breads, we found 352 molecular features fulfilling the same inclusion criteria as for the features with increased levels, including 69 identified metabolites (Table S1). 143 features (34 identified compounds) fulfilled the criteria in the wheat bread samples and 49 features (18 identified compounds) in both wheat and rye samples. The identified decreased metabolites in either rye or wheat sourdough included phenolic acids (caffeic, ferulic, *p*-coumaric and sinapic acid), five nucleosides involved in nucleic acid synthesis (adenosine, cytidine, guanosine, thymidine and uridine), nucleoside derivatives and nucleobases (adenine, cytosine, guanine, and thymine), amino acid–derived betaines (trimethyllysine and valine betaine), *myo*-inositol, two phosphatidylcholines [LysoPC(15:0) and PC(16:0/18:3)], mono- and polysaccharides, and oxidized fatty acids (shown for rye in Fig. 4C). There were differences between the wheat and rye sourdoughs: the two amino acid-derived betaines and most of the fatty acids, nucleobases and sugars fulfilled the inclusion criteria only in rye. As shown in Fig. 3, the levels of nucleosides and their derivatives and nucleobases were increased by yeast fermentation while the relative levels were low in both rye and wheat flours and sourdoughs.

We identified several compounds belonging to the major phytochemical classes known to exist in whole-grain cereals that were not significantly (*p* < 0.01) increased or decreased in their levels based on the chosen thresholds in sourdough fermented breads. Nine alkylresorcinols (17:0, 17:1, 19:1, 19:2, 21:1, and the levels of four oxidated alkylresorcinols) were significantly (*p* < 0.05) increased in the sourdough rye bread (between 1.15 and 3.49; Table S2). None of the levels of alkylresorcinols identified from the data were significantly changed in wheat sourdough. In total, twelve amino acid-derived betaines were identified from the data, many of them previously unreported from cereals (4-aminobutyric acid betaine, glutamic acid betaine, glutamine betaine, histidine betaine, isoleucine betaine, pipecolic acid betaine, and trimethyllysine). In addition to trimethyllysine and valine betaine, the levels of histidine betaine and trigonelline were significantly decreased in rye sourdough (FC −1.89 and −1.49, respectively) and isoleucine betaine increased in both rye and wheat sourdough (FC 3.57 and 2.97, respectively). The levels of detected lignans were generally too low for reliable identification; the only putatively identified lignan, buddlenol C, showed significant minor decrease in levels (*p* < 0.001, FC −1.27) in rye sourdough and significant minor increase in levels (*p* < 0.05, FC 1.15) in wheat sourdough.

## Discussion

The principal component analysis indicated that the most substantial difference in the metabolic profiles of the analyzed samples was between the flours and all the other samples (including the doughs and breads). Clear differences were seen between wheat and rye, between whole-grain and processed samples, and between rye sourdoughs and straight doughs, highlighting the significance of food processing in the final biochemical composition of the products. The effect of sourdough alone was considerable, especially in rye, where more than 700 molecular features had increased levels with the chosen inclusion criteria and over 350 decreased levels with high significance. These changes are likely contributing to the differences in the sensory properties and the potential metabolic effects of sourdough bread. Many of the observed changes in the metabolite levels were specific to rye sourdough; overall, sourdough fermentation of whole-grain wheat produced considerably less significantly changed metabolites and a less separated metabolic profile from yeast-fermented wheat dough and bread in the PCA. The main reason behind this is likely the different conditions chosen for the two sourdoughs, selected to represent widely consumed breads, with the higher temperature and longer fermentation time of rye sourdough allowing a more extensive metabolism. In contrast to rye, only mild acidity is accepted by consumers in wheat sourdough bread, thus requiring differences in the fermentation process^16^. The appearance of certain metabolites exclusively in rye sourdough may also be explained by the wider range of compounds in rye available for microbial metabolism, differences between wheat and rye flour matrices, the development of LAB ecology during the baking, and different endogenous enzymatic activity in the two species.

Sourdough fermentation considerably increased the levels of branched-chain amino acids leucine and isoleucine, BCAA metabolites as well as several small peptides containing BCAAs. This effect was more prominent in rye than wheat sourdough most likely due to intensive proteolysis in the acidic rye sourdough utilized in this study. As has been shown previously, the consumption of rye bread decreases the postprandial insulin response without a decrease in glucose response due to an unknown mechanism independent from the dietary fiber content of the bread^10,17^. BCAAs are known to activate the mTORC1 signaling pathway in skeletal muscle in a similar manner to exercise, and this in turn leads to uncoupling of the insulin signaling^18^. This may contribute to the decreased insulin response seen *in vivo*. However, the role of BCAAs in insulin metabolism is more complex than this, as their increased circulating levels are associated with obesity-related insulin resistance, possibly because of an overload of BCAA catabolism^19^. In a study by Moazzami *et al*., higher fasting concentrations of leucine and isoleucine were correlating with a higher insulin response after the intake of all types of study breads^20^. In contrast, phenylalanine and methionine, which were also among the significantly increased amino acids in sourdough rye in the current study, were the main metabolites associated with a lowered insulin response after 60 minutes of sourdough rye bread intake^21^. Few clinical studies on cereal products and their glycemic responses have included a comparison of fermented and unfermented bread. In a randomized cross-over trial, Johansson *et al*.^22^ found a lower insulin response for unfermented whole-grain rye crisp bread compared with yeast fermented bread and hypothesized that the effect could be partly explained by the BCAA levels increased by the yeast fermentation (up to 23%). The discrepancy in the effects of BCAAs in insulin response may be due to the difference in the role of moderate intake of dietary BCAAs and pathogenic metabolic pathways related to insulin resistance, manifesting as increased circulating BCAA levels. Leucic acid, a leucine metabolite, is known for its anti-catabolic effects on muscle tissue^23^; it remains unclear whether this compound or the other BCAA-related metabolites identified in the current study (2-hydroxyisovaleric acid, 2-isopropylmalic acid, 3-methyl-2-oxovaleric acid, and ketoleucine) could exert their own impact on the insulin response. A clinical study on the effect of sourdough fermented rye bread in insulin response, having yeast fermented rye as comparison, could further elucidate whether sourdough fermentation is one of the main contributors to the observed rye factor.

Several small peptides have been reported to possess potential antioxidant and antihypertensive activity^24,25^. These characteristics seem to be governed by the presence of certain amino acids in the peptide sequence; e.g. leucine may increase both the radical scavenging activity and ACE inhibition of the peptide^26^. Specifically, 28 peptides that had increased levels in sourdough (marked with an asterisk in Table S1) have been included in the database of antihypertensive peptides^27^ and VKL as antioxidant^24^.

The main phenolic acids present in rye and wheat – ferulic, caffeic, *p-*coumaric, and sinapic acid – had significantly lower levels in both rye and wheat sourdough. Correspondingly, the levels of several known microbial metabolites of phenolic acids^28,29^ were increased after sourdough fermentation, indicating that they were likely metabolized by the LAB strains. This observation is in agreement with *in vitro* studies, where various LAB strains were shown to metabolize all the main phenolic acids^30,31^. These microbial metabolites have different absorption and metabolic characteristics than their precursors^32^, which may have significance regarding the bioactivity of these compounds. Although rye and wheat are not abundant sources of flavonoids, we observed an increase in the levels of isorhamnetin and a corresponding decrease in the levels of isorhamnetin-3-*O*-hexoside, indicating the release of the flavonoid aglycone from its glycoside by the bacteria. The levels of most alkylresorcinols increased in rye sourdough. Since the increase was absent in wheat sourdough, the observation is most likely not originating from analytical factors, such as the relatively poor solubility of alkylresorcinols in commonly used extraction solvents. This result is in disagreement with previous studies, where alkylresorcinols have been reported to either decrease^33,34^ or exhibit only minor changes^35^ after sourdough fermentation. The increase of alkylresorcinol levels seen here in only sourdough rye might be explained by different capabilities of LAB strains to release or metabolize these compounds and the softer matrix in rye bran compared to wheat^34^.

Interestingly, nucleobases, nucleosides and their derivatives had lower levels in sourdough wheat and rye compared to yeast fermented samples. Since their relative levels were found to be low in flours as well, they are likely metabolites of yeast fermentation. Little is known about the biological significance of these compounds regarding dietary intake; among the few reported effects is that they may act as immunomodulators in infants receiving nucleosides in breast milk^36^. Four species of phosphatidylcholines had significantly changed levels in rye sourdough. The compounds with observed increase in their levels contain polyunsaturated fatty acids (PUFAs), likely linoleic acid and alpha- or gammalinolenic acid. The dietary intake of ω − 3 phosphatidylcholines has been shown to improve fatty acid and glucose metabolism in rats^37^, but on the other hand, the colonic microbial metabolism of the choline group in phosphatidylcholines may result in adverse cardiovascular effects^38^. We could not determine in the current study whether the identified phosphatidylcholine species contain ω − 3 or ω − 6 PUFAs; in addition, more research is needed to determine the association of polyunsaturated phosphatidylcholine intake with the risk of CVD and diabetes.

While non-targeted metabolomics is ideal for a wide-scale investigation of the metabolic profile of any biological sample, it is limited by the incomplete availability of spectral references for the reliable annotation of all statistically significant compounds, and this holds true especially for metabolite-rich sample matrices, such as the ones studied in this work. The high relative amount of unknowns may cause bias towards more well-known types of compounds, for which more reference data is available. Therefore, future targeted studies are warranted to further investigate the changes occurring in these metabolites as well as providing confirmation to the identifications. There is some uncertainty in annotating small peptides due to limitations in separating the signals originating from leucine and isoleucine and deducing the correct order of the amino acid residues in the peptide. Peptides larger than three amino acid residues have limited reference spectra available. The different fermentation conditions in wheat and rye sourdoughs inevitably affect the metabolic profiles observed in the current study; however, the differences in the profiles are relevant regarding the potential health implications, since the studied breads represent similar types of breads as normally consumed. The selection of LAB strains may affect the metabolic profiles or sourdough fermentation depending on the array of enzymes present in each strain. Here, we aimed to use one representative sourdough starter; studying the effect of the selection of strains on the same metabolic profiles is warranted for the future.

The non-targeted metabolomics approach provided wide insight into the metabolic profile of sourdough fermentation. We hypothesize that sourdough fermentation contributes to the beneficial health effects of whole grains by increasing the amount of several bioactive compounds, such as BCAAs, small peptides, and microbial phenolic acid metabolites, in the baked products. The effect is more profound in rye sourdough compared to wheat, likely attributable to the more extensive metabolism occurring in a typical rye sourdough and suggesting a potential contributor to the rye factor. The current study indicates the potential for future research to reveal the molecular basis of physiological signals caused by wholegrain bread intake, and in more general, of the observed protective effect of whole grains against non-communicable diseases.

## Materials and Methods

### Raw materials

Four different types of flours were used in the baking: endosperm wheat flour (manufacturer’s product code: V500P), wholegrain wheat flour (V1700), endosperm rye flour (R700) and wholegrain rye flour, coarse (R1800KA). All flours were obtained from Fazer Mill & Mixes, Finland. The nutritional composition of the flours is presented in Table 1.

**Table 1:** The content (as is) of protein, dietary fibre and fat in the flours as provided by the manufacturer.

	Endosperm wheat	Wholegrain wheat	Endosperm rye	Wholegrain rye
Protein	12.0	12.0	5.5	9.1
Dietary fibre	4.4	13.0	9.6	18.0
Fat	1.7	3.2	2.3	2.3

### Preparation of sourdoughs

The microbial starters, their levels and fermentation conditions used in the preparation of sourdoughs are presented in Table 2. The selected yeast (*Candida milleri*) and lactic acid bacteria (*Lactobacillus brevis* and *Lactobacillus plantarum*) are commonly found in both rye and wheat sourdoughs^39^. For the rye sourdough, a high proportion of starter and intensive fermentation conditions were selected to produce a high level of acidity, which is desirable for obtaining a good crumb structure in rye bread^40^. For the wheat sourdough, a lower level of starter and milder fermentation conditions were selected to produce a moderate acidity level, which has been related to improved wheat sourdough bread flavour and volume^16^. The microbial strains were obtained from the culture collection of VTT. The starters were prepared by refreshing the microbial strains twice in succession in general edible medium^41^ for 24 h at 30 °C. The cells were collected from the cultures by centrifugation and suspended in sterile water to obtain desired cell concentrations. Sourdoughs were prepared by mixing 1 kg of flour with 1.5 l of water and starter suspension by hand and incubating the mixture in a covered container. After the fermentation, a sample was taken from the sourdough for the analysis of microbial growth and a sample was frozen for a subsequent analysis of acidity. The viable counts of lactic acid bacteria were determined using plate count technique on MRS agar (Oxoid, Basingstoke, UK). Yeasts were enumerated on YM agar (Difco laboratories, Detroit, USA). Acidity of the sourdoughs (pH and total titratable acidity, TTA) was analysed according to a standard method^42^. The fresh sourdoughs were immediately used for bread baking.

**Table 2:** The microbial starters and their dosages (cfu/g sourdough in the beginning of the fermentation), the fermentation conditions, the acidity test results and the microbial count results (cfu/g) of the sourdoughs at the end of the fermentation.

Strain	Wholegrain wheat	Wholegrain rye
*Candida milleri* C-96250	10^6^	10^7^
*Lactobacillus brevis* E-95612	10^7^	10^8^
*Lactobacillus plantarum* E-78076	10^7^	10^8^
Fermentation time and temperature	12 h, 24 °C	20 h, 32 °C
pH	4.7	3.8
Total titratable acidity (TTA), ml	8	15
LAB count (end of fermentation)	10^9^	10^9^
Yeast count (end of fermentation)	10^7^	10^8^

### Preparation of breads

Six different breads were prepared by straight dough or by sourdough baking process at VTT Technical Research Centre of Finland Ltd., Espoo (Table 3). For each bread, two replicates were baked on separate days to account for possible changes in the fermentation process. The water content of the dough for each of the flours was determined by a farinograph and/or adjusted by test baking. In the sourdough breads, 33% of the total flour was sourdough fermented. The water content of the dough was the same for the sourdough breads and the corresponding straight dough breads. The wheat breads were moulded mechanically (by a conical rounder and a long moulder) and the rye breads by hand (due to dough stickiness). After moulding, the dough pieces were placed in fat-sprayed aluminium pans for proofing. One of the pans was left without spray fat. After proofing, a dough sample was taken from the pan without spray fat in order to avoid extra fat in the proven dough sample. After baking, the breads were left to cool down for 2–4 h before an analysis of volume and texture, except for the wholegrain rye breads, which were analysed one day after the baking because the breads were too sticky to be analysed on the baking day. A slice was cut from the middle of each bread, cut further into cubes of ca. 1 cm in size, and stored frozen in sealed plastic bags until the sample preparation for the analysis.

**Table 3:** The recipes (as percentage of flour weight) and the baking process of the breads.

	Straight dough breads				Sourdough breads	
	Endosperm wheat (WB)	Wholegrain wheat (WWB)	Endosperm rye (RB)	Wholegrain rye (WRB)	Wholegrain wheat (WWSB)	Wholegrain rye (WRSB)
Flour	100.0	100.0	100.0	100.0	66.7	66.7
Sugar	2.0	2.0	—	—	2.0	—
Sourdough	—	—	—	—	83.3	83.3
*of which flour*	—	—	—	—	*33.3*	*33.3*
*of which water*	—	—	—	—	*50.0*	*50.0*
Water	61.0	70.0	68.0	76.0	20.0	26.0
Yeast	3.0	3.0	3.0	3.0	3.0	3.0
Salt	1.2	1.2	1.2	1.2	1.2	1.2
Mixing	2 min slow + 4 min fast					
Resting	20 min 28 °C/80% RH		60 min 28 °C/80% RH		20 min 28 °C/80% RH	60 min 28 °C/80% RH
Dough piece weight	400 g		500 g		400 g	500 g
Moulding	mechanically		by hand		mechanically	by hand
Resting	8 min		—		8 min	—
Proofing	50 min 37 °C/8% RH		60 min 37 °C/80% RH		50 min 37 °C/80% RH	60 min 37 °C/80% RH
Steaming in oven	15 s					
Baking	225 °C 20 min		240 °C 10 min + 220 °C 35 min		225 °C 20 min	240 °C 10 min + 220 °C 35 min

### Sample preparation

Samples were obtained from each type of bread (*n* = 6), dough (*n* = 6) and flour (*n* = 4). The frozen samples (−80 °C) were cryoground into fine powder using a tissue homogenizer (TissueLyser II, Qiagen, Hilden, Germany). The grinding frequency was set at 20 s^−1^ and the duration at 40 s. The ground sample was inspected visually, and the procedure was repeated in the case of any unground pieces. Approximately 100 mg of each sample was weighed into Eppendorf tubes; at this stage, four technical replicates were taken from the flour samples and three replicates from the dough and bread samples into separate tubes. The metabolite extraction solvent was produced by mixing HPLC gradient grade methanol, Milli-Q water and 80% formic acid using a MeOH:H_2_O:HCOOH ratio of 80:19.9:0.1 v/v/v. The solution had a pH of 3.6. The extraction solvent was added into the tubes in a ratio of 300 µl to 100 mg of sample. The tubes were vortexed, sonicated for 15 min, vortexed again, and centrifuged for 10 min at 13,000 rpm. The supernatant was collected and filtered (Acrodisc CR 13 mm syringe filter with 0.2 µm PTFE membrane). The filtrate was then transferred to HPLC sample vials.

### LC–MS/MS analysis

The liquid chromatography–mass spectrometry was performed on a 1290 Infinity Binary UPLC coupled with a 6540 UHD Accurate-Mass Q-TOF (Agilent Technologies, Santa Clara, CA, USA) as described previously by Hanhineva *et al*.^43^ Briefly, a Zorbax Eclipse XDB-C18 column was used for the reversed-phase separation and an Aqcuity UPLC BEH amide column (Waters, Milford, MA, USA) for the HILIC separation. After each chromatographic separation, the ionization was carried out using jet stream electrospray ionization (ESI) in the positive and negative mode, yielding four data files per sample. The collision energies for the MS/MS analysis were chosen as 10, 20 and 40 V, for compatibility with the spectral databases.

### Statistical analysis and compound identification

The molecular features were extracted from the data by using MassHunter Qualitative Analysis version 7.0 (Agilent Technologies) and the biostatistical analysis was performed in Mass Profiler Professional version 2.2 (Agilent Technologies), as described previously^34^. The raw values of the peak areas were used in determining the relative levels of the compounds in different samples. The *p* values were calculated using one-way ANOVA and the fold changes as the ratio of the average raw peak areas of sourdough fermented samples to yeast fermented samples, using a negative inverse value in case of a ratio below 1 (level decrease). After the statistical analysis, filtering was performed in Microsoft Excel to extract the list of statistically significant peaks for each comparison. Fold change was set at ≥2 (overexpression in sourdough samples), *p* value at <0.01, RSD at ≤30% (variation within sample group), and average signal intensity (in the sample group being compared) at ≥200 000. Principal component analysis (PCA) was performed in SIMCA version 14 (Umetrics AB) using Pareto scaling. Hierarchical Clustering method in Multiple Array Viewer version 4.9.0 (TM4 Software Suite) was used to create the heat map of the significant identified compounds.

The accurate masses, retention times, and MS/MS fragmentation patterns of the detected statistically significant compounds were used in comparison with an in-house standard library, previously published literature, and freely available MS spectral databases. The tentative annotation of compounds based on spectral database searches was performed in MS-DIAL^44^. For some of the compounds with no spectral database matches, tentative IDs were given based on *in silico* generated MS/MS spectra acquired from molecular database entries using MS-FINDER software^45^. For the identification of small peptides, fragment motifs specific for amino acid residues were determined from the identifications based on MS/MS data and used in the further identification process. The fragment motifs observed in at least two peptides and used in the identifications are presented in Table 4.

**Table 4:** Proposed MS/MS fragment motifs and their tentative structures associated with amino acid residues of small peptides in mass spectra from LC–MS with electrospray ionization in the positive mode (ESI+).

Fragment *m/z*	Amino acid	Tentative fragment structure
44.050	(unspecific)	
55.053	valine	unknown
56.049	(unspecific)	
60.044	serine	side chain
60.055	arginine	guanidine group
69.071	leucine, isoleucine	branched hydrocarbon chain
70.065	(unspecific)	
72.080	valine	branched hydrocarbon chain
74.057	threonine	side chain
84.080	lysine, leucine, isoleucine	side chain
86.095	leucine, isoleucine	branched hydrocarbon chain
101.072	threonine	side chain
110.071	histidine	side chain
116.070	proline, arginine	molecular ion (proline)
117.032	valine	molecular ion
120.078	phenylalanine	side chain
130.048	glutamine	side chain
136.078	tyrosine	side chain
147.077	glutamine	molecular ion
175.120	arginine	molecular ion

### Data availability

The datasets generated and analyzed during the current study are available from the corresponding author on reasonable request.

## Electronic supplementary material

Supplementary information
