# Supplementary material for: Metabolic profiling of sourdough fermented wheat and rye bread
Source: Sci Rep. 2018 Apr 9;8:5684. doi: 10.1038/s41598-018-24149-w (PMC5890289; doi:10.1038/s41598-018-24149-w)
Supplement: Supplementary file 1 — Supplementary information [file 41598_2018_24149_MOESM1_ESM.docx]

**Metabolic profiling of sourdough fermented wheat and rye bread**

Ville M Koistinen^a^*, Outi Mattila^b^, Kati Katina^c^, Kaisa Poutanen^b^, Anna-Marja Aura^b^, Kati Hanhineva^a^

^a^ University of Eastern Finland, Institute of Public Health and Clinical Nutrition, P.O. Box 1627, FI-70211 Kuopio, Finland

^b^ VTT Technical Research Centre of Finland, P.O. Box 1000, Tietotie 2, Espoo, FI-02044 VTT, Finland

^c^ University of Helsinki, Department of Food and Environmental Sciences, P.O. Box 66 (Agnes Sjöbergin katu 2), FI-00014 University of Helsinki, Finland.

*Corresponding author (Email: ville.m.koistinen@uef.fi)

**SUPPLEMENTARY MATERIAL CAPTIONS**

**Table S1.** The identified and putatively identified compounds that had significantly (*p* < 0.01) changed levels (FC ≥ 2) in sourdough rye and/or wheat bread. FC = fold change for sourdough vs. yeast fermented bread; negative value represents the decrease in levels in sourdough. Statistically significant fold changes are marked with asterisks: * *p* < 0.05, ** *p* < 0.01, *** *p* < 0.001. ID level 2+ indicates that the experimental MS/MS data has been compared to *in silico* generated MS/MS data. Amino acid residues: A = alanine, D = aspartic acid, E = glutamic acid, F = phenylalanine, G = glycine, H = histidine, I = isoleucine, K = lysine, L = leucine, N = asparagine, P = proline, Q = glutamine, R = arginine, S = serine, T = threonine, V = valine, W = tryptophan, Y = tyrosine.

**Table S2.** The putatively identified alkylresorcinols (not included among the most significant metabolites) and their fold changes in rye and wheat sourdough. Statistically significant fold changes are marked with asterisks: * *p* < 0.05, ** *p* < 0.01, *** *p* < 0.001.

**Table S1**

| Tentative ID | Compound group | [M–H]^–^ | [M+H]^+^ | Column | Ion mode | Fragments | CID (V) | RT (min) | FC (rye) | FC (wheat) | ID level | Reference |
| --- | --- | --- | --- | --- | --- | --- | --- | --- | --- | --- | --- | --- |
| L-asparagine | amino acid | 131.0460 |  | HILIC | negative | 41.999 (100), 72.009 (14) | 20 | 6.20 | 8.0*** | –1.1 | 1 | standard |
| L-asparagine | amino acid |  | 133.0616 | HILIC | positive | 74.024 (100), 43.018 (43), 46.029 (38) | 20 | 6.20 | 5.1*** | –1.4** | 1 | standard |
| L-citrulline | amino acid |  | 176.1035 | HILIC | positive | 70.066 (100), 113.071 (21) | 20 | 6.33 | 4.5*** | 5.0*** | 1 | standard |
| L-glutamic acid | amino acid | 146.0455 |  | HILIC | negative | 102.057 (100), 74.024 (6), 128.034 (6) | 20 | 6.35 | 2.7*** | 1.5*** | 1 | standard |
| L-homocitrulline | amino acid |  | 173.0919 | HILIC | positive | 127.087 (100), 173.094 (68), 44.050 (50), 83.061 (38), 56.049 (37), 98.058 (28) | 20 | 5.02 | –2.2*** | –1.3* | 2 | MS-DIAL |
| L-isoleucine | amino acid |  | 132.1023 | HILIC | positive | 44.050 (100), 56.051 (59), 57.058 (49), 41.038 (30), 70.029 (21) | 40 | 3.91 | 10.7*** | 1.3*** | 1 | standard |
| L-leucine | amino acid |  | 132.1025 | HILIC | positive | 43.054 (100), 86.096 (52), 69.070 (44), 30.035 (38), 44.050 (32) | 20 | 3.68 | 17.0*** | 1.7*** | 1 | standard |
| L-lysine | amino acid |  | 147.1136 | HILIC | positive | 84.080 (100), 67.054 (7), 130.087 (2) | 20 | 7.11 | 2.8*** | 1.1 | 1 | standard |
| L-methionine | amino acid |  | 150.0586 | HILIC | positive | 56.049 (100), 61.011 (47), 133.032 (29), 87.026 (21), 77.007 (12) | 20 | 4.36 | 13.1*** | 1.8*** | 1 | standard |
| L-ornithine | amino acid |  | 133.0976 | HILIC | positive | 70.066 (100) | 20 | 7.17 | 5.9*** | 1.3** | 1 | standard |
| L-phenylalanine | amino acid | 164.0710 |  | RP | negative | 103.056 (100), 147.047 (50), 72.010 (28) | 20 | 1.95 | 8.7*** | 1.9*** | 1 | standard |
| L-phenylalanine | amino acid |  | 166.0868 | HILIC | positive | 120.082 (100), 93.069 (13), 77.038 (8), 103.054 (8) | 20 | 3.62 | 16.2*** | 1.6*** | 1 | standard |
| L-phenylalanine | amino acid | 164.0712 |  | HILIC | negative | 103.055 (100), 119.994 (44), 161.042 (28) | 20 | 3.64 | 9.7*** | 1.3*** | 1 | standard |
| L-tryptophan | amino acid |  | 205.0975 | HILIC | positive | 146.060 (100), 118.066 (72), 144.081 (23), 188.072 (14), 170.060 (13) | 20 | 3.82 | 3.0*** | –1.1** | 1 | standard |
| *N*-alpha-acetyl-L-arginine | amino acid |  | 217.1303 | HILIC | positive | 70.066 (100), 158.090 (46), 116.073 (34) | 20 | 5.62 | –6.4** | 1.5*** | 2 | MS-DIAL |
| saccharopine (isomer) | amino acid |  | 277.1403 | HILIC | positive | 84.081 (100), 130.087 (19), 231.149 (4), 213.117 (4) | 20 | 7.68 | –3.2*** | -1.0 | 2 | MS-DIAL |
| saccharopine (isomer) | amino acid |  | 259.1291 | HILIC | positive | 84.081 (100), 213.123 (17) | 20 | 6.87 | 3.1*** | 2.4*** | 2 | MS-DIAL |
| 2-amino-3-hydroxybenzoic acid | amino acid metabolite |  | 136.0401 | RP | positive | 80.051 (100), 136.040 (96), 44.998 (42), 63.023 (34), 53.003 (21) | 10 | 4.41 | –4.3*** | 2.3 | 2 | MS-DIAL |
| 2-hydroxyisovaleric acid | amino acid metabolite | 117.0554 |  | RP | negative | 71.050 (100) | 20 | 2.70 | 4.0*** | 5.0*** | 1 | standard |
| 2-isopropylmalic acid | amino acid metabolite | 175.0610 |  | HILIC | negative | 115.040 (100), 175.060 (95), 113.061 (42), 85.066 (32), 157.050 (15) | 10 | 0.83 | –2.4*** | –1.2 | 2 | MS-DIAL |
| 3-methyl-2-oxovaleric acid | amino acid metabolite | 129.0553 |  | RP | negative | 44.999 (100) | 20 | 3.20 | –17.6*** | –5.0*** | 1 | standard |
| 3-phenyllactic acid | amino acid metabolite | 165.0552 |  | RP | negative | 119.050 (100), 103.056 (86), 147.044 (53), 72.992 (35), 101.042 (32), 91.057 (25) | 20 | 4.49 | 27.6*** | 15.5*** | 1 | standard |
| 3-phenyllactic acid | amino acid metabolite | 165.0554 |  | HILIC | negative | 103.056 (100), 119.050 (75), 72.993 (65), 147.045 (55) | 20 | 0.70 | 35.2*** | 15.2*** | 1 | standard |
| 4-hydroxyphenyllactic acid | amino acid metabolite | 181.0502 |  | RP | negative | 135.046 (100), 119.050 (57), 134.035 (52), 72.992 (32) | 20 | 2.72 | 13.9*** | 24.0*** | 2 | METLIN, MS-DIAL |
| 4-hydroxyphenyllactic acid | amino acid metabolite | 181.0501 |  | HILIC | negative | 119.052 (100), 134.036 (98), 72.990 (43), 135.046 (21) | 20 | 1.03 | 35.8** | 121*** | 1 | standard |
| indole-3-lactic acid | amino acid metabolite | 204.0664 |  | RP | negative | 158.061 (100), 116.051 (90), 142.066 (49), 128.053 (38) | 20 | 4.50 | 5.8*** | 5.4*** | 2 | MS-DIAL |
| ketoleucine | amino acid metabolite | 129.0554 |  | RP | negative | 70.125 (100) | 20 | 3.45 | –3.9*** | –2.7*** | 1 | standard |
| leucic acid | amino acid metabolite | 131.0708 |  | RP | negative | 85.066 (100), 131.071 (60) | 10 | 4.25 | 21.6*** | 15.2*** | 2 | MS-DIAL |
| leucic acid | amino acid metabolite | 131.0711 |  | HILIC | negative | 85.067 (100), 131.069 (51), 71.024 (41), 69.035 (27) | 10 | 0.67 | 12.4*** | 11.7*** | 2 | MS-DIAL |
| tyramine | amino acid metabolite |  | 138.0923 | HILIC | positive | 121.067 (100), 77.038 (84), 103.054 (84), 93.070 (42), 91.055 (24) | 20 | 1.33 | ∞*** | ∞*** | 1 | standard |
| 2-benzoxazolinone | benzoxazinoid | 134.0242 |  | RP | negative | 134.025 (100), 41.998 (47), 78.035 (22), 91.018 (19) | 20 | 4.43 | –3.2*** | 1.8*** | 2 | DOI:10.1021/jf103612u |
| 2-benzoxazolinone (isomer) | benzoxazinoid | 134.0246 |  | RP | negative | 134.026 (100), 41.998 (66), 78.033 (22) | 20 | 3.63 | 16.8** | 15.6* | 2 | DOI:10.1021/jf103612u |
| acetylcholine | betaine |  | 146.1180 | HILIC | positive | 87.044 (100), 43.019 (84), 60.081 (5) | 20 | 0.80 | 43.0 | 191*** | 1 | standard |
| trimethyllysine | betaine |  | 189.1600 | HILIC | positive | 84.082 (100), 130.087 (15), 60.081 (13), 144.139 (4) | 20 | 6.76 | –2.1*** | –1.0 | 2 | DOI:10.1002/jms.2029 |
| valine betaine | betaine |  | 160.1338 | HILIC | positive | 60.082 (100), 59.074 (47), 58.066 (40), 160.133 (30) | 20 | 1.86 | –3.5** | 1.0 | 2 | DOI:10.1002/mnfr.201500066 |
| 2-hydroxyvaleric acid | carboxylic acid | 117.0556 |  | HILIC | negative | 71.051 (100), 117.056 (45), 44.998 (24) | 20 | 0.74 | 4.1*** | 4.2*** | 2 | MS-DIAL |
| citric acid | carboxylic acid | 191.0195 |  | HILIC | negative | 87.010 (100), 111.008 (79), 85.030 (53), 191.054 (50) | 20 | 5.86 | –2.1*** | 1.2 | 2 | standard without RT |
| glucoheptonic acid | carboxylic acid | 225.0610 |  | HILIC | negative | 59.014 (100), 71.013 (27), 89.025 (17), 44.999 (8), 113.023 (8) | 20 | 4.97 | –3.3*** | –3.0*** | 2 | MS-DIAL |
| lactic acid | carboxylic acid | 89.0242 |  | HILIC | negative | 43.019 (100), 41.004 (16), 89.025 (7) | 20 | 1.31 | 10.7*** | 8.1*** | 1 | standard |
| lactic acid (peak 2) | carboxylic acid | 89.0243 |  | HILIC | negative | 43.019 (100) | 20 | 1.41 | 6.5*** | 7.2*** | 1 | standard |
| lipoic acid | carboxylic acid | 205.0349 |  | HILIC | negative | 53.040 (100), 99.008 (83), 81.035 (82), 72.996 (63), 125.023 (58) | 20 | 5.03 | 2.4*** | 1.8*** | 2+ | MS-FINDER |
| malic acid | carboxylic acid | 133.0137 |  | HILIC | negative | 115.004 (100), 133.014 (33), 71.014 (30), 72.992 (15) | 10 | 4.72 | –1.8*** | –4.7*** | 2 | MS-DIAL |
| *N*-acetylglycine | carboxylic acid | 116.0347 |  | HILIC | negative | 74.024 (100), 72.045 (22), 116.035 (12) | 10 | 2.48 | 2.7*** | 5.7*** | 1 | standard |
| nicotinic acid | carboxylic acid |  | 124.0398 | HILIC | positive | 124.040 (100), 80.051 (30), 78.034 (20), 53.039 (17) | 20 | 5.51 | –4.4*** | –1.8*** | 2 | METLIN |
| *N*-phenylacetylglycine | carboxylic acid |  | 194.0822 | RP | positive | 89.038 (100), 78.045 (44), 65.038 (27), 117.032 (22) | 20 | 3.38 | –2.3*** | 2.5 | 2+ | MS-FINDER |
| tridecanedioic acid | carboxylic acid |  | 227.1655 | RP | positive | 56.025 (100), 71.049 (92), 95.051 (86), 79.055 (85), 41.039 (76) | 10 | 7.82 | –2.2** | 1.2 | 2+ | MS-FINDER |
| 1-monopalmitin | fatty acid |  | 331.2860 | RP | positive | 43.055 (100), 57.070 (90), 41.039 (48), 67.054 (19) | 40 | 10.55 | –1.2 | –2.3** | 2 | MS-DIAL |
| 9-HPODE | fatty acid | 311.2234 |  | RP | negative | 293.213 (100), 125.097 (40), 185.119 (32), 149.097 (30), 57.035 (22) | 20 | 9.60 | ∞*** | nd | 1 | standard |
| 9-HpOTrE | fatty acid |  | 293.2126 | RP | positive | 95.048 (100), 67.053 (71), 55.018 (60), 83.050 (29), 81.069 (27), 131.085 (20) | 10 | 9.35 | –4.0*** | 1.9*** | 2 | MS-DIAL |
| 9-HpOTrE | fatty acid | 309.2074 |  | RP | negative | 171.105 (100), 137.100 (87), 301.167 (48), 43.882 (38) | 40 | 9.28 | –4.0*** | 1.1 | 2 | MS-DIAL |
| 9-HpOTrE | fatty acid | 309.2081 |  | RP | negative | 171.101 (100), 291.202 (61), 137.098 (31), 183.101 (27) | 20 | 8.78 | –4.0** | –1.2 | 2 | MS-DIAL |
| 9-hydroxyoctadecadienoic acid | fatty acid | 295.2282 |  | RP | negative | 277.217 (100), 295.228 (61), 171.102 (43), 195.139 (16) | 20 | 9.61 | –7.7*** | 1.4*** | 2 | MS-DIAL |
| 9-OxoOTrE | fatty acid | 291.1971 |  | RP | negative | 185.118 (100), 125.096 (82), 121.103 (48) | 20 | 9.36 | 1.3*** | 2.0*** | 2 | MS-DIAL |
| corchorifatty acid | fatty acid | 327.2184 |  | RP | negative | 239.163 (100), 171.100 (46), 195.141 (37), 137.096 (33), 209.1209 (27), 185.121 (26) | 20 | 7.98 | –2.4*** | –2.0* | 2+ | MS-FINDER |
| DiHODE | fatty acid |  | 313.2389 | RP | positive | 55.019 (100), 67.056 (85), 81.070 (85), 41.040 (84) | 20 | 9.11 | –5.5*** | –1.2 | 2+ | MS-FINDER |
| DiHOME | fatty acid |  | 315.2543 | RP | positive | 43.055 (100), 81.071 (88), 79.056 (55), 67.055 (50), 93.069 (38), 55.054 (32) | 20 | 9.07 | –2.1*** | –1.2 | 2 | MS-DIAL |
| dodecanedioic acid | fatty acid |  | 213.1496 | RP | positive | 93.070 (100), 41.039 (93), 67.056 (60), 77.039 (60), 149.1311 (44) | 10 | 7.81 | –2.5*** | –2.0** | 2 | MS-DIAL |
| epoxyoctadecanoic acid | fatty acid |  | 279.2335 | RP | positive | 67.055 (100), 95.086 (61), 81.071 (41), 41.039 (23), 119.085 (16) | 20 | 9.61 | –8.4*** | 1.3** | 2 | MS-DIAL |
| HpEDE | fatty acid |  | 323.2596 | RP | positive | 105.073 (100), 79.061 (96), 55.056 (94), 68.057 (88), 43.053 (80), 119.087 (62) | 40 | 9.89 | –3.8*** | 1.8*** | 2 | MS-DIAL |
| hydroxyhexadecanoic acid | fatty acid | 271.2285 |  | RP | negative | 271.230 (100), 225.224 (15), 99.083 (14), 155.144 (10) | 20 | 9.74 | –2.1*** | 1.6** | 2+ | MS-FINDER |
| oxidized fatty acid | fatty acid | 311.2234 |  | RP | negative | 311.224 (100), 293.213 (29), 171.103 (22), 195.139 (17) | 20 | 9.04 | –3.0*** | 1.3*** | 2 | MS-DIAL |
| oxo-octadecadienoic acid | fatty acid |  | 295.2285 | RP | positive | 43.055 (100), 67.056 (94), 55.055 (92), 91.054 (73) | 20 | 7.81 | –3.0*** | –1.3** | 2 | MS-DIAL |
| oxo-octadecadienoic acid | fatty acid |  | 295.2284 | RP | positive | 55.054 (100), 67.054 (54), 79.053 (29), 93.070 (25), 43.054 (20), 107.085 (14) | 20 | 9.04 | –4.1*** | –1.7* | 2 | MS-DIAL |
| oxo-octadecanoic acid | fatty acid | 297.2447 |  | RP | negative | 297.245 (100), 185.119 (75), 279.236 (14) | 20 | 9.79 | 38.1*** | 4.4*** | 2+ | MS-FINDER |
| PUFA | fatty acid |  | 307.2650 | RP | positive | 67.054 (100), 81.069 (47), 95.085 (29), 55.053 (28) | 20 | 10.29 | 1.3** | 2.2*** | 2 | MS-DIAL |
| trans-EKODE | fatty acid |  | 311.2235 | RP | positive | 95.049 (100), 93.070 (60), 43.054 (52), 294.217 (46), 87.081 (39), 133.104 (34) | 10 | 7.99 | –4.3*** | –1.5*** | 2 | MS-DIAL |
| adenine | nucleobase |  | 136.0613 | RP | positive | 119.037 (100), 65.014 (77), 92.026 (49), 53.014 (39) | 20 | 1.42 | –84.7** | –1.9 | 2 | MS-DIAL |
| cytosine | nucleobase |  | 112.0513 | HILIC | positive | 95.025 (100), 68.013 (23), 67.030 (21) | 20 | 2.00 | –212*** | –1.6*** | 1 | standard |
| guanine | nucleobase |  | 152.0575 | RP | positive | 152.056 (100), 135.029 (75), 110.033 (30) | 10 | 1.50 | –157** | –1.8* | 2 | MS-DIAL |
| guanine | nucleobase |  | 152.0570 | HILIC | positive | 135.031 (100), 110.036 (30) | 20 | 2.63 | –∞*** | –1.4* | 1 | standard |
| methyladenine | nucleobase |  | 150.0783 | HILIC | positive | 150.079 (100), 133.051 (27), 57.046 (11), 123.067 (10), 106.041 (9), 82.041 (8) | 20 | 1.95 | –3.3** | 1.8*** | 2 | MS-DIAL |
| nucleobase | nucleobase |  | 127.0509 | RP | positive | 54.035 (100), 82.028 (74), 127.049 (39), 45.988 (37), 68.966 (34), 84.050 (28) | 10 | 2.01 | –37.7** | –2.2*** | 2 | MS-DIAL |
| thymine | nucleobase | 125.0351 |  | RP | negative | 106.871 (100), 41.997 (93), 78.960 (88) | 40 | 1.38 | ∞*** | 216*** | 1 | standard |
| uridine | nucleobase | 243.0622 |  | HILIC | negative | 41.999 (100), 110.026 (92), 243.063 (60), 152.040 (42) | 20 | 1.40 | –5.5*** | –4.2 | 1 | standard |
| 1-methyladenosine | nucleoside |  | 282.1203 | HILIC | positive | 150.078 (100) | 20 | 4.17 | –4.8*** | –2.4*** | 1 | standard |
| 2'-deoxyadenosine | nucleoside |  | 252.1105 | RP | positive | 136.062 (100), 119.036 (24), 92.025 (23) | 20 | 1.48 | –∞*** | –1.8*** | 1 | standard |
| 5-methylcytidine | nucleoside |  | 258.1093 | HILIC | positive | 126.067 (100), 109.039 (36), 83.061 (28), 81.044 (22), 56.049 (11) | 40 | 2.56 | –19.1*** | –7.9*** | 2 | MS-DIAL |
| 5-methyldeoxycytidine | nucleoside |  | 242.1157 | HILIC | positive | 126.062 (100), 109.037 (55), 83.057 (22), 56.047 (20) | 40 | 1.69 | –∞*** | ∞*** | 2+ | MS-FINDER |
| adenosine | nucleoside |  | 268.1057 | RP | positive | 136.059 (100), 119.033 (16), 268.102 (8), 94.038 (3) | 10 | 1.42 | –189** | –2.4*** | 1 | standard |
| adenosine | nucleoside | 266.0894 |  | RP | negative | 134.046 (100), 266.089 (12), 183.665 (8) | 10 | 1.44 | –2.9 | –3.1*** | 1 | standard |
| adenosine | nucleoside |  | 268.0982 | HILIC | positive | 136.062 (100), 119.035 (19), 57.034 (8) | 40 | 1.30 | –∞*** | –∞* | 1 | standard |
| cytidine | nucleoside |  | 244.0943 | HILIC | positive | 112.051 (100), 95.024 (49), 69.044 (25) | 40 | 3.10 | –6.3*** | –4.0*** | 1 | standard |
| deoxycytidine | nucleoside |  | 228.0988 | HILIC | positive | 112.051 (100), 95.023 (90), 69.044 (43), 67.029 (25) | 40 | 1.99 | –9.4 | –2.0*** | 1 | standard |
| deoxycytidine [2M+H]^+^ | nucleoside |  | 455.1897 | HILIC | positive | 112.051 (100), 455.237 (72), 228.097 (48) | 10 | 1.99 | –11.7*** | –2.2*** | 1 | standard |
| deoxyguanosine | nucleoside | 266.0892 |  | RP | negative | 150.041 (100), 108.022 (12), 133.015 (11), 266.086 (10) | 20 | 1.60 | –∞*** | –2.4*** | 2 | MS-DIAL |
| guanosine | nucleoside | 282.0845 |  | RP | negative | 150.042 (100), 133.014 (10), 108.021 (9) | 20 | 1.49 | –3.3*** | –4.0*** | 1 | standard |
| guanosine | nucleoside |  | 284.1004 | RP | positive | 152.058 (100), 135.031 (50), 110.035 (12) | 10 | 1.47 | –119** | –4.1*** | 1 | standard |
| guanosine | nucleoside |  | 284.0997 | HILIC | positive | 152.056 (100), 135.029 (6) | 20 | 3.94 | –2.9*** | –4.4*** | 1 | standard |
| guanosine | nucleoside | 282.0839 |  | HILIC | negative | 150.042 (100), 133.017 (51), 108.021 (13) | 20 | 3.98 | –4.0*** | –5.2*** | 1 | standard |
| methyladenosine | nucleoside |  | 282.1210 | RP | positive | 136.061 (100), 119.036 (44), 55.018 (36), 94.040 (20) | 40 | 1.92 | –62.8*** | –6.2*** | 2+ | MS-FINDER |
| methylguanosine | nucleoside |  | 298.1153 | HILIC | positive | 149.046 (100), 166.072 (48), 124.050 (21) | 40 | 4.47 | –16.2*** | –8.7*** | 2 | MS-DIAL |
| *N,N*-dimethylguanosine | nucleoside |  | 312.1316 | RP | positive | 180.086 (100), 110.035 (75), 135.028 (29), 55.028 (23), 71.013 (22) | 20 | 2.43 | –58.2*** | –5.0*** | 2+ | MS-FINDER |
| thymidine | nucleoside | 241.0828 |  | RP | negative | 41.998 (100) | 10 | 2.02 | –4.6*** | –2.1*** | 1 | standard |
| thymidine | nucleoside | 241.0829 |  | HILIC | negative | 41.999 (100), 151.111 (57) | 10 | 0.83 | –5.7*** | –3.0*** | 1 | standard |
| 1-linoleoylglycerol | other |  | 355.2870 | RP | positive | 67.055 (100), 55.055 (95), 95.086 (88), 81.070 (72), 109.101 (49), 41.038 (42) | 20 | 10.38 | 2.5** | 1.4** | 2 | MS-DIAL |
| delta-guanidinovaleric acid | other |  | 160.1087 | HILIC | positive | 160.107 (100), 87.044 (36), 104.070 (32), 60.081 (17) | 10 | 2.37 | –4.4** | 1.1** | 2+ | MS-FINDER |
| DG(15:0/18:4) | other |  | 575.4687 | RP | positive | 135.115 (100), 43.053 (80), 205.191 (76), 81.069 (54), 121.099 (50) | 20 | 10.94 | 2.7*** | 1.8 | 2+ | MS-FINDER |
| glucosylglycerol | other | 253.0930 |  | RP | negative | 44.998 (100) | 40 | 1.23 | 1.3* | 2.2*** | 2+ | MS-FINDER |
| glycerophosphoglycerol | other | 245.0433 |  | HILIC | negative | 78.959 (100), 152.996 (62), 171.007 (33) | 20 | 6.21 | 3.3*** | 1.7*** | 2+ | MS-FINDER |
| GPE(16:0) | other |  | 454.2948 | HILIC | positive | 313.274 (100), 44.050 (90), 454.236 (37), 155.010 (19), 204.103 (17) | 20 | 1.78 | –8.8*** | 1.4** | 2 | MS-DIAL |
| histidinol | other |  | 142.0980 | HILIC | positive | 81.045 (100), 95.061 (19), 60.045 (15), 72.061 (8), 107.060 (8) | 20 | 5.61 | –8.6*** | –1.4 | 1 | standard |
| levoglucosan | other | 161.0451 |  | HILIC | negative | 85.030 (100), 71.012 (92), 57.035 (79), 59.015 (51), 43.019 (43), 67.020 (38) | 20 | 3.76 | 2.1** | –1.5 | 2 | MS-DIAL |
| LPG(16:0) | other | 483.2754 |  | HILIC | negative | 255.234 (100), 483.274 (66), 245.448 (8), 226.507 (5) | 20 | 1.33 | –2.6*** | 1.0 | 2 | MS-DIAL |
| LysoPE(18:1) | other | 478.2952 |  | RP | negative | 281.249 (100), 214.049 (3) | 20 | 10.29 | –5.9*** | 1.3* | 2 | MS-DIAL |
| *N*-acetylspermidine | other |  | 188.1765 | HILIC | positive | 72.082 (100), 100.076 (54) | 20 | 6.16 | 22.1*** | 62.0*** | 2+ | MS-FINDER |
| *N*-deoxyfructosyl-leucine | other |  | 294.1557 | RP | positive | 88.039 (100), 138.055 (53), 53.039 (43), 230.140 (42) | 20 | 1.54 | –325*** | 1.3*** | 2+ | MS-FINDER |
| 7-hydroxy-4-(methoxymethyl)coumarin | other phenolic |  | 207.0662 | RP | positive | 63.025 (100), 76.031 (54), 105.069 (44), 65.039 (40), 91.052 (32) | 20 | 4.58 | –2.6*** | –7.5* | 2 | MS-DIAL |
| isorhamnetin | other phenolic | 315.0511 |  | RP | negative | 63.024 (100), 108.022 (50), 107.014 (49), 83.014 (48), 94.006 (34) | 20 | 6.91 | 2.8*** | 4.1*** | 1 | standard |
| isorhamnetin-3-*O*-hexoside | other phenolic |  | 625.1778 | RP | positive | 317.066 (100), 107.048 (21), 359.079 (17), 127.038 (16), 85.026 (14) | 20 | 5.57 | –2.0*** | –1.2** | 2 | MS-DIAL |
| phenylethanolamine | other phenolic |  | 120.0816 | RP | positive | 51.023 (100), 77.040 (70), 103.054 (34), 50.016 (17) | 20 | 1.95 | 5.7*** | 1.7** | 2 | MS-DIAL |
| vanillin | other phenolic |  | 153.0554 | RP | positive | 65.040 (100), 39.023 (73), 110.036 (36), 53.038 (15) | 20 | 3.92 | –3.1*** | –1.4*** | 2 | MS-DIAL |
| AL* | peptide |  | 203.1398 | RP | positive | 86.096 (100), 132.101 (33), 44.050 (31), 65.925 (26) | 20 | 2.13 | 2.9*** | –1.2*** | 2 | MS-DIAL |
| AP* | peptide |  | 187.1081 | HILIC | positive | 70.065 (100), 44.049 (38), 116.0704 (22) | 20 | 5.59 | 4.4 | –2.4*** | 2 | MS-DIAL |
| DLLS | peptide | 445.2305 |  | RP | negative | 357.179 (100), 129.068 (40), 401.207 (31) | 20 | 4.62 | 8.5*** | 4.1 | 2+ | MS-FINDER |
| EIV | peptide | 358.1990 |  | RP | negative | 229.156 (100), 314.209 (68), 211.146 (61), 128.036 (53) | 20 | 2.56 | 19.0*** | 8.6*** | 2+ | MS-FINDER |
| EVV | peptide | 344.1827 |  | HILIC | negative | 102.058 (100), 215.141 (94), 128.036 (87), 146.046 (86), 300.191 (78), 344.187 (73), 197.130 (58) | 20 | 5.59 | 11.4*** | 1.4** | 2+ | MS-FINDER |
| EW | peptide |  | 334.1408 | RP | positive | 188.072 (100), 84.044 (90), 146.060 (34), 101.037 (31) | 20 | 3.60 | –3.3 | –2.3*** | 2 | METLIN |
| FLLL | peptide |  | 527.3201 | RP | positive | 86.096 (100), 242.151 (27) | 20 | 4.32 | 80.8** | nd | 2 | METLIN |
| GL* | peptide | 187.1082 |  | HILIC | negative | 130.088 (100), 73.042 (51), 143.120 (47) | 20 | 5.11 | 1.8*** | –3.2** | 2 | MS-DIAL |
| GVL | peptide |  | 288.1928 | RP | positive | 72.081 (100), 129.100 (44), 55.054 (22), 86.097 (16), 157.097 (12), 132.098 (7) | 10 | 3.58 | 12.2*** | 1.5** | 2 | METLIN |
| HK* | peptide | 282.1553 |  | HILIC | negative | 89.025 (100), 43.019 (13) | 20 | 1.41 | 21.4*** | 17.0*** | 2+ | MS-FINDER |
| HL* | peptide |  | 269.1617 | HILIC | positive | 110.071 (100), 83.061 (7), 178.132 (6), 86.097 (4) | 20 | 5.59 | 2.5*** | –1.5*** | 2 | MS-DIAL |
| IF* | peptide |  | 279.1715 | RP | positive | 86.096 (100), 69.069 (95), 44.048 (25), 120.077 (13) | 20 | 4.05 | 6.4*** | 1.6*** | 2 | MS-DIAL |
| II* | peptide |  | 245.1870 | RP | positive | 86.096 (100), 69.070 (40), 30.033 (22), 44.049 (21), 57.057 (18) | 20 | 3.25 | 12.4*** | –1.2** | 2 | MS-DIAL |
| IL* | peptide |  | 245.1869 | RP | positive | 86.096 (100), 69.071 (38), 43.056 (16), 44.049 (14) | 20 | 3.57 | 9.6*** | 1.1 | 2 | MS-DIAL |
| ITR | peptide |  | 389.2398 | RP | positive | 86.096 (100), 72.080 (36), 74.059 (22), 56.050 (18) | 20 | 2.37 | ∞*** | nd | 2 | MS-DIAL |
| LA | peptide |  | 203.1323 | RP | positive | 86.093 (100), 70.071 (46), 56.048 (42) | 20 | 1.48 | ∞*** | nd | 2 | MS-DIAL |
| LAS | peptide | 288.1561 |  | HILIC | negative | 74.026 (100), 169.096 (52), 244.130 (43), 88.041 (37), 200.138 (32) | 20 | 5.62 | 14.0*** | 1.4** | 2+ | MS-FINDER |
| LE* | peptide |  | 261.1459 | RP | positive | 84.046 (100), 56.050 (94), 41.039 (73), 86.096 (39) | 40 | 2.23 | 1.1 | –3.7*** | 2 | METLIN |
| LF* | peptide |  | 279.1712 | RP | positive | 43.054 (100), 103.053 (40), 86.100 (31), 44.049 (29), 120.079 (26) | 20 | 4.27 | 13.7*** | 2.4*** | 2 | MS-DIAL |
| LG* | peptide |  | 189.1243 | RP | positive | 86.099 (100), 44.050 (20), 70.068 (14) | 20 | 2.16 | 3.0*** | –2.1*** | 2 | MS-DIAL |
| LGL* | peptide |  | 302.2090 | RP | positive | 86.097 (100), 143.118 (15), 69.069 (14) | 20 | 4.33 | 7.6*** | 1.3** | 2 | METLIN |
| LGR | peptide |  | 345.2251 | HILIC | positive | 345.223 (100), 86.095 (74), 215.111 (34), 253.165 (34), 246.153 (22) | 20 | 6.67 | 6.0*** | 1.0 | 2 | MS-DIAL |
| LL* | peptide |  | 245.1874 | RP | positive | 44.050 (100), 86.098 (80) | 20 | 3.81 | 6.5*** | 2.2*** | 2 | MS-DIAL |
| LL* | peptide |  | 245.1864 | HILIC | positive | 86.0972 (100), 41.038 (6), 69.070 (3), 132.099 (2) | 20 | 1.79 | 9.2*** | 1.4*** | 2 | MS-DIAL |
| LL* | peptide | 243.1708 |  | HILIC | negative | 130.088 (100), 199.181 (90), 115.085 (58), 99.924 (52) | 20 | 1.82 | 13.9*** | 1.4*** | 2 | MS-DIAL |
| LL* | peptide | 243.1710 |  | RP | negative | 243.173 (100), 130.088 (40), 199.184 (24) | 10 | 3.57 | 11.1*** | 1.4*** | 2 | MS-DIAL |
| LLA | peptide |  | 316.2242 | RP | positive | 86.097 (100), 157.138 (27), 44.049 (25), 41.038 (23) | 10 | 4.27 | 20.3*** | 1.9*** | 2+ | MS-FINDER |
| LLL* | peptide |  | 358.2714 | HILIC | positive | 86.097 (100), 199.181 (30) | 20 | 1.26 | 49.5*** | 5.6*** | 2 | METLIN |
| LLL* | peptide |  | 358.2717 | RP | positive | 86.097 (100), 69.071 (12) | 20 | 5.27 | 12.3*** | 5.1*** | 2 | METLIN |
| LLL* | peptide | 356.2560 |  | RP | negative | 130.088 (100), 356.258 (70), 312.264 (63), 225.159 (23) | 20 | 5.21 | 41.1*** | 4.1 | 2+ | MS-FINDER |
| LLL* | peptide |  | 358.2715 | RP | positive | 86.097 (100), 69.071 (12) | 20 | 5.21 | 27.9*** | 3.1*** | 2 | METLIN |
| LLQ | peptide |  | 373.2455 | RP | positive | 86.097 (100), 84.044 (42), 147.077 (28), 130.049 (21), 69.070 (16) | 20 | 2.51 | 124** | nd | 2 | METLIN |
| LP* | peptide |  | 457.3037 | RP | positive | 70.066 (100), 56.964 (12), 44.050 (8) | 20 | 2.39 | 18.6*** | 4.7 | 2 | MS-DIAL |
| LQ* | peptide | 258.1455 |  | HILIC | negative | 88.040 (100), 58.663 (20), 141.105 (16) | 20 | 5.33 | 11.9*** | 2.0 | 2+ | MS-FINDER |
| LQL | peptide |  | 745.4801 | RP | positive | 86.095 (100), 242.152 (34), 131.117 (25), 30.034 (17) | 10 | 4.57 | 158*** | –∞ | 2 | METLIN |
| LR | peptide |  | 288.2036 | HILIC | positive | 86.096 (100), 175.120 (88), 70.066 (44), 69.070 (32), 114.103 (28), 288.202 (26) | 20 | 6.35 | 43.0** | 3.6*** | 2 | MS-DIAL |
| LS | peptide | 217.1191 |  | HILIC | negative | 129.103 (100), 173.092 (80), 74.025 (63), 155.083 (40) | 20 | 5.58 | 11.1*** | 1.2*** | 2+ | MS-FINDER |
| LT | peptide | 231.1345 |  | HILIC | negative | 143.118 (100), 187.110 (65), 74.025 (63) | 20 | 5.26 | 6.9*** | 2.6*** | 2+ | MS-FINDER |
| LV* | peptide |  | 231.1709 | RP | positive | 86.096 (100), 44.051 (63), 70.064 (40) | 20 | 2.41 | 9.9*** | 1.1 | 2 | MS-DIAL |
| LV* | peptide | 229.1555 |  | RP | negative | 229.157 (100), 116.070 (28), 210.950 (20), 185.167 (15) | 10 | 2.41 | 12.1*** | 1.1 | 2 | MS-DIAL |
| LV* | peptide |  | 231.1714 | RP | positive | 86.096 (100), 57.053 (33) | 20 | 2.60 | 5.1*** | –1.4*** | 2 | MS-DIAL |
| LVG | peptide | 286.1770 |  | RP | negative | 129.104 (100), 74.026 (70), 211.148 (40), 242.185 (34) | 20 | 2.11 | 68.3*** | 14.6*** | 2+ | MS-FINDER |
| LVG | peptide |  | 288.1931 | RP | positive | 86.097 (100), 72.080 (59), 41.039 (28), 70.065 (23) | 20 | 2.11 | 155** | 3.4 | 2 | METLIN |
| LVG | peptide | 286.1769 |  | HILIC | negative | 185.165 (100), 130.088 (59), 155.081 (44), 286.178 (35), 73.042 (32) | 20 | 3.93 | 20.9*** | 1.3* | 2+ | MS-FINDER |
| LVR* | peptide |  | 387.2717 | HILIC | positive | 387.271 (100), 175.120 (81), 185.166 (48), 72.080 (31), 158.098 (20), 112.086 (17), 156.112 (17), 352.234 (16), 257.160 (13) | 20 | 6.10 | 34.6*** | 1.0 | 2 | MS-DIAL |
| LVS | peptide | 316.1874 |  | RP | negative | 130.088 (100), 286.175 (91), 143.120 (57), 155.082 (53), 242.183 (51) | 20 | 3.89 | 30.8*** | 3.4*** | 2+ | MS-FINDER |
| LVS | peptide | 316.1876 |  | HILIC | negative | 61.989 (100), 74.026 (8), 254.191 (7) | 20 | 5.02 | 17.5*** | 1.5** | 2+ | MS-FINDER |
| LVS | peptide |  | 318.2023 | RP | positive | 72.079 (100), 86.094 (92), 44.047 (23) | 20 | 2.02 | 14.0*** | 1.1 | 2 | METLIN |
| LVV | peptide |  | 330.2400 | RP | positive | 72.082 (100), 86.098 (75), 185.163 (44), 55.055 (15), 178.079 (15) | 20 | 3.85 | 41.2*** | 2.1*** | 2 | METLIN |
| LVV | peptide |  | 330.2398 | RP | positive | 86.097 (100), 72.082 (87), 213.160 (29), 185.164 (28), 69.073 (23), 55.055 (23), 44.050 (18) | 10 | 4.05 | 14.2*** | 1.7*** | 2 | METLIN |
| NVK | peptide |  | 360.2142 | RP | positive | 78.082 (100), 55.053 (53), 132.102 (37) | 10 | 3.49 | 9.8*** | 2.1*** | 2 | MS-DIAL |
| PF* | peptide |  | 263.1401 | RP | positive | 70.064 (100), 56.049 (16), 128.050 (16) | 20 | 2.83 | 20.9*** | 2.1*** | 2 | MS-DIAL |
| PL* | peptide | 227.1398 |  | HILIC | negative | 59.014 (100), 114.055 (48), 82.064 (32), 165.053 (28), 70.067 (28) | 20 | 3.94 | 5.1*** | –2.2*** | 2+ | MS-FINDER |
| PL* | peptide |  | 229.1554 | RP | positive | 70.066 (100) | 20 | 2.39 | 18.4*** | 3.2*** | 2 | MS-DIAL |
| PL* | peptide |  | 229.1553 | HILIC | positive | 70.065 (100) | 20 | 3.06 | 12.3*** | 2.2*** | 2 | MS-DIAL |
| PV | peptide |  | 215.1389 | HILIC | positive | 72.081 (100), 70.064 (15) | 20 | 4.65 | 2.8*** | –3.6*** | 2 | MS-DIAL |
| QEP | peptide |  | 355.1621 | HILIC | positive | 116.070 (100), 129.066 (75), 45.035 (34), 70.066 (27), 212.105 (19), 355.097 (17), 84.043 (15) | 20 | 5.47 | 13.1*** | 2.3*** | 2 | MS-DIAL |
| QEP | peptide | 353.1470 |  | RP | negative | 238.084 (100), 114.056 (90), 126.019 (70) | 20 | 2.27 | 17.8*** | 1.9*** | 2 | MS-DIAL |
| QLLP | peptide |  | 470.2979 | RP | positive | 86.096 (100), 101.069 (63), 84.043 (49), 227.098 (22), 83.060 (22) | 20 | 3.69 | ∞*** | –∞*** | 2+ | MS-FINDER |
| QQR | peptide | 429.2100 |  | RP | negative | 429.212 (100), 145.062 (81) | 20 | 1.96 | 35.3*** | 1.5 | 2 | MS-DIAL |
| QSLL | peptide |  | 460.2776 | RP | positive | 86.097 (100), 201.121 (65), 173.127 (62), 260.162 (58), 130.052 (38), 147.077 (35), 60.044 (25) | 20 | 3.57 | ∞*** | ∞ | 2 | METLIN |
| SL* | peptide |  | 219.1344 | RP | positive | 60.044 (100), 86.094 (70), 173.126 (47), 160.038 (28) | 10 | 1.98 | 14.9*** | –1.4 | 2 | MS-DIAL |
| SLL | peptide | 330.2043 |  | RP | negative | 130.086 (100), 256.202 (71), 300.191 (55), 199.181 (28), 83.026 (22) | 20 | 4.23 | 109** | 1.9 | 2+ | MS-FINDER |
| SLL | peptide | 330.2029 |  | HILIC | negative | 74.025 (100), 242.184 (68), 129.104 (59), 286.179 (47) | 20 | 4.84 | 20.3*** | 1.0 | 2+ | MS-FINDER |
| SLL | peptide |  | 332.2195 | RP | positive | 86.098 (100), 60.045 (75), 44.050 (66), 173.129 (59) | 20 | 4.24 | 74.4*** | –2.3* | 2 | METLIN |
| tetrapeptide | peptide |  | 496.2641 | RP | positive | 86.096 (100), 200.076 (62), 72.081 (53), 167.094 (48), 139.094 (37), 185.092 (34), 74.060 (34) | 20 | 2.65 | ∞*** | nd | 3 | exact mass, fragmentation pattern |
| TL | peptide |  | 233.1504 | RP | positive | 56.049 (100), 74.057 (59), 86.097 (39) | 20 | 2.20 | 2.5*** | –1.3*** | 2 | MS-DIAL |
| TVL | peptide | 330.2036 |  | RP | negative | 130.087 (100), 286.178 (28), 242.187 (22), 185.166 (19), 155.081 (18) | 20 | 3.56 | 9.8*** | 3.9*** | 2 | MS-DIAL |
| TVL | peptide |  | 332.2197 | RP | positive | 55.054 (100), 72.081 (69), 56.049 (56), 74.063 (42), 101.072 (40), 173.131 (34) | 20 | 3.56 | 6.2*** | 2.9*** | 2 | MS-DIAL |
| TVL | peptide | 330.2040 |  | HILIC | negative | 130.087 (100), 286.181 (49), 242.188 (48), 330.203 (23) | 20 | 3.86 | ∞*** | –∞* | 2 | MS-DIAL |
| VALQ | peptide |  | 430.2675 | RP | positive | 86.097 (100), 130.050 (56), 44.050 (33), 110.072 (22), 147.076 (19), 84.085 (18), 102.055 (14), 72.081 (12) | 20 | 3.64 | ∞*** | nd | 2 | METLIN |
| VF* | peptide |  | 265.1560 | RP | positive | 55.053 (100), 72.080 (72) | 20 | 3.35 | 3.9*** | 1.3*** | 2 | MS-DIAL |
| VIT | peptide |  | 332.2188 | RP | positive | 86.097 (100), 72.082 (52), 55.054 (15), 69.070 (14), 44.051 (12), 185.167 (11) | 20 | 2.61 | 11.8*** | –5.3** | 2 | METLIN |
| VKL | peptide |  | 359.2670 | RP | positive | 55.054 (100), 72.082 (73), 56.049 (55), 84.080 (38) | 20 | 2.90 | 6.1*** | 4.0*** | 2 | METLIN |
| VL* | peptide | 229.1551 |  | HILIC | negative | 130.088 (100), 185.164 (32), 116.072 (31) | 20 | 2.49 | 13.7*** | 2.2*** | 2 | MS-DIAL |
| VL* | peptide |  | 231.1708 | HILIC | positive | 72.081 (100), 86.096 (38) | 20 | 2.45 | 8.9*** | 1.5*** | 2 | MS-DIAL |
| VL* | peptide |  | 231.1710 | RP | positive | 72.083 (100), 55.056 (85) | 10 | 2.89 | 4.5*** | 1.2*** | 2 | MS-DIAL |
| VLE | peptide |  | 360.2139 | RP | positive | 86.097 (100), 72.080 (77), 70.065 (50), 150.066 (43) | 20 | 2.56 | ∞*** | ∞*** | 2 | METLIN |
| VLL | peptide |  | 344.2563 | RP | positive | 72.081 (100), 86.097 (90), 44.049 (8) | 20 | 4.99 | 18.3*** | 3.3*** | 2 | METLIN |
| VLL | peptide |  | 344.2559 | RP | positive | 86.097 (100), 69.071 (72), 72.080 (55), 185.170 (32), 168.085 (17) | 20 | 4.81 | 10.1*** | 2.4*** | 2 | METLIN |
| VLQ | peptide |  | 359.2291 | HILIC | positive | 147.076 (100), 130.048 (86), 86.096 (60), 185.164 (49), 84.045 (18) | 20 | 5.45 | 105*** | –∞ | 2 | METLIN |
| VLR | peptide |  | 387.2631 | RP | positive | 70.064 (100), 85.028 (94), 60.055 (69), 61.028 (69) | 40 | 1.51 | ∞*** | nd | 2 | MS-DIAL |
| VLV | peptide |  | 330.2398 | RP | positive | 86.095 (100), 72.080 (30), 69.070 (18), 84.081 (14) | 20 | 4.20 | 14.3*** | 2.6*** | 2 | METLIN |
| VPL | peptide |  | 328.2241 | RP | positive | 86.097 (100), 72.081 (69), 116.069 (47), 70.065 (46) | 10 | 3.59 | 4.9*** | 1.6*** | 2 | METLIN |
| VPN | peptide |  | 329.1833 | RP | positive | 70.066 (100), 72.081 (67), 196.061 (27), 44.049 (18) | 20 | 1.86 | 15.3*** | 1.6 | 2 | METLIN |
| VR* | peptide |  | 274.1879 | HILIC | positive | 72.081 (100), 175.120 (80), 60.057 (47), 116.073 (27), 130.099 (22) | 20 | 6.66 | 5.8*** | 1.8*** | 2 | MS-DIAL |
| VSL | peptide |  | 318.2011 | RP | positive | 72.081 (100), 86.095 (17), 159.111 (17), 132.101 (15), 118.063 (7) | 20 | 3.89 | 24.6*** | 4.1 | 2 | METLIN |
| VV* | peptide | 215.1398 |  | HILIC | negative | 215.141 (100), 116.070 (22), 171.151 (20) | 20 | 3.53 | 13.1*** | –1.5*** | 2 | MS-DIAL |
| VVP | peptide |  | 314.2087 | RP | positive | 72.081 (100), 55.055 (40), 70.066 (34), 116.070 (14) | 20 | 2.83 | 18.7*** | 1.4** | 2 | METLIN |
| VVR | peptide |  | 373.2567 | HILIC | positive | 72.082 (100), 55.055 (16), 158.093 (11), 60.057 (11) | 40 | 6.36 | 42.4*** | 1.7*** | 2 | MS-DIAL |
| YL* | peptide |  | 295.1661 | RP | positive | 136.078 (100), 91.053 (65), 132.016 (56), 86.095 (50), 96.043 (50) | 10 | 3.18 | 4.7*** | 1.4*** | 2 | METLIN |
| YP* | peptide |  | 279.1349 | RP | positive | 91.060 (100), 70.067 (89), 90.048 (28), 119.050 (23), 136.076 (19) | 20 | 2.27 | 1.0 | –3.8*** | 2 | MS-DIAL |
| YQK | peptide |  | 438.2400 | RP | positive | 147.043 (100), 438.235 (89), 204.097 (62), 91.051 (45), 292.198 (38) | 10 | 4.50 | –2.6 | 4.7*** | 2 | MS-DIAL |
| YV* | peptide |  | 281.1507 | RP | positive | 136.077 (100), 150.081 (30), 45.034 (24) | 10 | 2.03 | 8.4*** | 1.8*** | 2 | MS-DIAL |
| caffeic acid | phenolic acid | 179.0341 |  | RP | negative | 135.046 (100), 179.037 (33), 44.999 (19) | 10 | 3.41 | –3.7* | –2.3*** | 1 | standard |
| dihydrofcaffeic acid | phenolic acid | 181.0504 |  | RP | negative | 137.061 (100), 135.047 (70), 43.001 (59), 93.002 (58), 59.012 (44) | 10 | 3.22 | ∞*** | ∞*** | 2 | METLIN |
| dihydroferulic acid | phenolic acid | 195.0664 |  | RP | negative | 136.052 (100), 121.028 (33), 44.999 (12), 135.046 (10) | 20 | 4.37 | 136*** | 165** | 2 | DOI:10.1016/j.jchromb.2009.10.006; DOI:10.1016/j.jpba.2013.10.009 |
| dihydrosinapic acid | phenolic acid | 225.0767 |  | RP | negative | 151.043 (100), 123.046 (72), 225.080 (M-H, 44), 166.065 (35), 150.031 (35), 165.056 (33) | 20 | 4.51 | 54.3** | ∞*** | 2 | DOI:10.1016/j.nut.2011.06.005 |
| ferulic acid | phenolic acid | 193.0506 |  | RP | negative | 134.038 (100), 178.028 (10) | 20 | 4.52 | –3.9*** | –10.0*** | 1 | standard |
| feruloylagmatine | phenolic acid |  | 613.3507 | RP | positive | 89.039 (100), 177.055 (95), 145.028 (88), 117.033 (65) | 20 | 2.92 | ∞*** | 1.4 | 2 | DOI:10.1016/j.phytochem.2011.08.021 |
| galloyl hexose | phenolic acid | 331.0671 |  | RP | negative | 125.024 (100), 331.067 (93), 168.004 (63) | 20 | 1.75 | –2.0** | 1.3* | 2+ | MS-FINDER |
| hexadecyl ferulate | phenolic acid |  | 419.3171 | RP | positive | 43.054 (100), 57.071 (98), 71.086 (56), 149.022 (37) | 20 | 11.34 | –1.5 | 2.1*** | 2+ | MS-FINDER |
| *N1,N10*-diferuloylspermidine | phenolic acid |  | 498.2574 | HILIC | positive | 177.054 (100), 145.029 (25), 234.113 (10), 322.216 (7) | 40 | 1.35 | –∞*** | –∞*** | 2+ | MS-FINDER |
| *p*-coumaric acid | phenolic acid | 163.0396 |  | RP | negative | 119.050 (100), 93.035 (11), 163.037 (4) | 20 | 4.16 | –2.5*** | –10.6* | 1 | standard |
| *p*-coumaroylputrescine | phenolic acid |  | 235.1397 | HILIC | positive | 147.044 (100), 235.119 (21), 110.070 (18), 119.050 (16) | 20 | 1.81 | ∞*** | 4.4 | 2+ | MS-FINDER |
| sinapic acid | phenolic acid | 223.0610 |  | RP | negative | 193.014 (100), 149.023 (51), 121.030 (43), 93.034 (31), 164.047 (29), 163.038 (27), 135.045 (20) | 20 | 4.59 | –2.1*** | –4.1*** | 1 | standard |
| sinapic acid | phenolic acid |  | 225.0772 | RP | positive | 65.039 (100), 89.037 (34), 53.004 (22), 121.027 (21) | 20 | 4.58 | –2.7 | –5.3 | 2 | MS-DIAL |
| LysoPC(15:0) | phosphatidylcholine |  | 482.3240 | HILIC | positive | 104.1074 (100), 184.073 (99), 482.325 (51), 86.096 (8), 124.998 (6) | 20 | 1.23 | –2.5*** | –1.0 | 2 | MS-DIAL |
| PC(16:0/18:3) | phosphatidylcholine |  | 756.5557 | RP | positive | 184.075 (100), 756.554 (23) | 20 | 11.98 | –11.5*** | –1.4* | 2 | MS-DIAL lipidomics |
| PC(18:2/18:2) | phosphatidylcholine |  | 782.5735 | RP | positive | 184.075 (100), 86.098 (11), 125.000 (7) | 40 | 12.08 | 2.4*** | 1.1 | 1 | standard |
| PC(18:2/18:2) | phosphatidylcholine | 826.5633 |  | RP | negative | 279.235 (100) | 40 | 12.11 | 2.3*** | 1.1 | 1 | standard |
| PC(18:2/18:2) | phosphatidylcholine |  | 782.5715 | HILIC | positive | 184.073 (100), 86.097 (5), 125.000 (2) | 40 | 0.69 | 2.1** | –1.1 | 2 | MS-DIAL |
| PC(18:2/18:3) | phosphatidylcholine |  | 780.5569 | RP | positive | 184.075 (100), 780.555 (23) | 20 | 11.81 | 2.8*** | 1.0 | 2 | MS-DIAL lipidomics |
| PC(18:3/18:2) | phosphatidylcholine | 824.5465 |  | RP | negative | 279.233 (100), 277.218 (83), 44.999 (16) | 40 | 11.84 | 2.8*** | –1.1 | 2 | MS-DIAL |
| D-lyxose | sugar | 149.0450 |  | HILIC | negative | 59.015 (100), 99.276 (40), 71.014 (38) | 20 | 2.53 | 2.4*** | 2.0*** | 2 | MS-DIAL |
| D-ribonolactone (peak 1) | sugar | 165.0399 |  | HILIC | negative | 75.009 (100), 165.040 (50), 72.993 (33), 105.019 (30) | 10 | 5.97 | 2.2*** | 2.0* | 2 | MS-DIAL |
| D-ribonolactone (peak 2) | sugar | 165.0401 |  | HILIC | negative | 75.009 (100), 59.015 (38), 56.999 (30) | 20 | 5.72 | 2.7*** | 3.9*** | 2 | MS-DIAL |
| lactulose | sugar | 683.2291 |  | HILIC | negative | 161.047 (100), 179.057 (57), 101.025 (21), 89.025 (15), 143.035 (12) | 20 | 6.26 | –10.0*** | 1.5 | 2 | MS-DIAL |
| maltose / cellobiose | sugar | 341.1101 |  | HILIC | negative | 101.025 (100), 59.015 (98), 73.031 (92), 89.026 (85) | 20 | 6.35 | –7.4*** | –1.6* | 1 | standard |
| maltotriose | sugar | 503.1623 |  | HILIC | negative | 179.055 (100), 101.024 (95), 161.045 (54), 89.023 (43), 221.066 (32), 143.035 (31) | 20 | 7.25 | –2.2*** | 1.1* | 2 | MS-DIAL |
| *myo*-inositol | sugar | 89.0238 |  | HILIC | negative | 59.015 (100), 157.896 (13), 71.013 (9), 83.013 (8) | 20 | 6.25 | –4.9*** | –1.1* | 2 | standard (without MS/MS) |
| raffinose | sugar | 503.1622 |  | HILIC | negative | 179.057 (100), 503.165 (75), 89.024 (63), 161.047 (45), 221.069 (31) | 20 | 7.16 | –3.5*** | –1.6* | 1 | standard |
| stachyose | sugar |  | 689.2132 | HILIC | positive | 689.2134 (100), 365.104 (90), 527.158 (57) | 40 | 7.46 | 1.1 | –2.6*** | 2 | MS-DIAL |
| stachyose | sugar | 665.2156 |  | HILIC | negative | 161.048 (100), 443.142 (55), 545.172 (40), 341.108 (36) | 20 | 8.04 | –3.1*** | 1.3 | 1 | standard |
| stachyose | sugar | 665.2158 |  | HILIC | negative | 89.024 (100), 113.025 (67), 59.014 (42), 161.045 (40), 101.026 (36) | 40 | 7.61 | 2.2** | 1.2*** | 1 | standard |
| sucrose | sugar | 341.1097 |  | HILIC | negative | 89.025 (100), 59.015 (98), 71.015 (40), 101.025 (38), 119.036 (37), 113.025 (30) | 20 | 5.96 | 2.5*** | –1.8 | 1 | standard |
| sugar | sugar | 339.1304 |  | RP | negative | 59.014 (100), 113.024 (54), 339.128 (38), 235.082 (36), 161.046 (34) | 20 | 1.75 | –3.8*** | 2.7*** | 2 | MS-DIAL |
| sugar | sugar | 209.0663 |  | HILIC | negative | 85.030 (100), 57.035 (65), 59.015 (56) | 20 | 5.76 | 2.7*** | 2.0*** | 2 | MS-DIAL |
| sugar | sugar |  | 360.1513 | HILIC | positive | 127.039 (100), 85.029 (88), 145.049 (41) | 20 | 5.94 | ∞*** | nd | 2 | MS-DIAL |
| β-D-glucose | sugar |  | 383.1169 | HILIC | positive | 203.054 (100) | 20 | 4.96 | –∞*** | –4.6*** | 2 | MS-DIAL |

**Table S2**

| Tentative ID | [M–H]^–^ | Column | Ion mode | Fragments | CID (V) | RT (min) | FC (rye) | FC (wheat) | ID level | Reference |
| --- | --- | --- | --- | --- | --- | --- | --- | --- | --- | --- |
| alkylresorcinol C15:0 | 319.2642 | RP | negative | 319.266 (100), 277.249 (11), 122.035 (7) | 20 | 10.76 | 1.21 | nd | 2 | DOI:10.1080/10408398.2015.1016477 |
| alkylresorcinol C17:0 | 347.2961 | RP | negative | 41.004 (100), 81.033 (52), 303.308 (50), 305.289 (40), 347.289 (33), 122.041 (23), 135.045 (16) | 40 | 11.06 | 1.57* | 1.16 | 2 | DOI:10.1080/10408398.2015.1016477 |
| alkylresorcinol C17:1 | 345.2805 | RP | negative | 41.003 (100), 122.037 (72), 303.264 (42), 81.035 (32), 345.275 (27), 135.044 (26), 149.057 (10) | 40 | 10.84 | 1.39** | 1.17 | 2 | DOI:10.1016/j.foodres.2016.06.027 |
| alkylresorcinol C19:0 | 375.3271 | RP | negative | 333.319 (100), 41.004 (91), 81.034 (64), 375.330 (23), 122.036 (23) | 40 | 11.37 | 1.68 | 1.17 | 2 | DOI:10.1080/10408398.2015.1016477 |
| alkylresorcinol C19:1 | 373.3113 | RP | negative | 41.004 (100), 81.035 (30), 373.317 (26), 135.044 (13), 265.197 (12), 122.037 (12), 225.003 (11) | 40 | 11.11 | 1.74** | 1.03 | 2 | DOI:10.1016/j.foodres.2016.06.027 |
| alkylresorcinol C19:2 | 371.2963 | RP | negative | 41.003 (100), 371.293 (42), 135.045 (41), 81.035 (23), 122.037 (21), 149.062 (20), 329.288 (19) | 40 | 10.92 | 1.45** | 1.09 | 2 | DOI:10.1016/j.foodres.2016.06.027 |
| alkylresorcinol C21:1 | 401.3427 | RP | negative | 401.343 (100), 359.330 (70), …, 81.035 (34) | 40 | 11.43 | 1.91** | 1.01 | 2 | DOI:10.1016/j.foodres.2016.06.027 |
| oxidated alkylresorcinol | 361.2750 | RP | negative | 123.045 (100), 81.034 (84), 278.409 (9), 79.054 (6) | 40 | 10.55 | 1.15* | nd | 3 | fragmentation pattern, exact mass |
| oxidated alkylresorcinol | 389.3072 | RP | negative | 123.045 (100), 81.035 (55) | 40 | 10.87 | 1.94*** | 1.24 | 2 | DOI:10.1016/j.foodres.2016.06.027 |
| oxidated alkylresorcinol | 415.3209 | RP | negative | 123.045 (100), 81.035 (50), 122.036 (4) | 40 | 10.93 | 3.49* | 2.02 | 2 | DOI:10.1016/j.foodres.2016.06.027 |
| oxidated alkylresorcinol | 417.3377 | RP | negative | 123.044 (100), 81.034 (23) | 40 | 11.15 | 1.94** | 0.95 | 2 | DOI:10.1016/j.foodres.2016.06.027 |
